# Polyphenol co-pigments enhanced the antioxidant capacity and color stability of blue honeysuckle juice during storage

**DOI:** 10.1016/j.fochx.2024.101848

**Published:** 2024-09-20

**Authors:** Yifan Geng, Kaojia Cui, Na Ding, Houping Liu, Junwei Huo, Xiaonan Sui, Yan Zhang

**Affiliations:** a Heilongjiang Green Food Science Research Institute, Northeast Agricultural University, Harbin 150030, China; b Key Laboratory of Biology and Genetic Improvement of Horticultural Crops (Northeast Region), Ministry of Agriculture and Rural Affairs, Northeast Agricultural University, Harbin 150030, China; c National-Local Joint Engineering Research Center for Development and Utilization of Small Fruits in Cold Regions, Northeast Agricultural University, Harbin 150030, China; d College of Horticulture and Landscape Architecture, Northeast Agricultural University, Harbin 150030, China; e College of Food Science, Northeast Agricultural University, Harbin 150030, China

**Keywords:** Co-pigments, Color protection, Polyphenols, Blue honeysuckle, Food processing

## Abstract

The study aimed to assess the impact of incorporating five co-pigments (gallic acid, quercetin, rutin, catechin, and epigallocatechin gallate (EGCG)) on the color stability of blue honeysuckle juice (BHJ). Additionally, it sought to determine the influence of varying proportions of anthocyanins in an accelerated test (light at 40 °C for 24 d). Results indicated that the addition of polyphenol co-pigments effectively mitigated the thermal degradation of anthocyanins, enhancing color saturation and antioxidant capacity of BHJ. Notably, quercetin, rutin, catechin, and EGCG exhibited superior efficacy compared to gallic acid. FTIR analysis revealed non-covalent complex formation between co-pigments and anthocyanins, including hydrogen bonds and van der Waals forces, thereby shielding them from degradation. HPLC-ESI-QTOF-MS^2^ identified 15 anthocyanins and 39 non-anthocyanin polyphenols. Addition of co-pigments effectively curbed anthocyanin degradation, thus stabilizing juice system. Consequently, judicious incorporation of co-pigments holds promise as a technology for enhancing the color quality and stability of BHJ during processing.

## Highlights

• Polyphenol co-pigments prevent BHJ anthocyanin degradation during storage.
• Hydrogen bonding is the main factor contributing to the improved anthocyanin stability.
• Flavonoid-anthocyanins co-pigmentation is more effective than that of phenolic acids.
• A total of 54 polyphenols was identified and quantified in blue honeysuckle juice (BHJ).
• Polyphenol co-pigmentation produces BHJ with excellent sensory quality and nutrition.

## Graphical abstract

**Figure:**
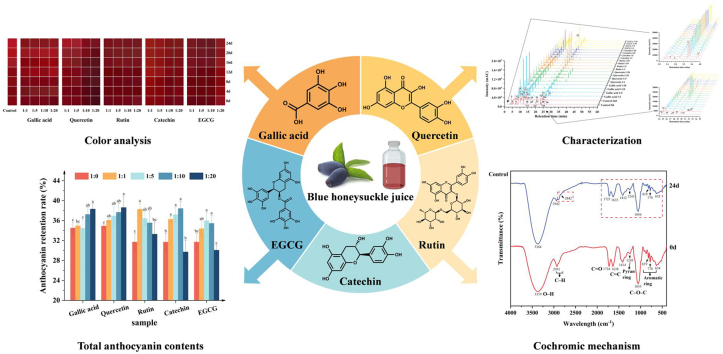

## 1 Introduction

Blue honeysuckle (*Lonicera caerulea* L.) is found in the north temperate regions. Renowned for its dual role as both a medicinal herb and a culinary delight, it has earned the moniker “king of the third generation of small berries.” Blue honeysuckle gained recognition by being included in the list of new foods (2015/2283 EU) in 2018, thereby receiving authorization for sale as a traditional food from many countries under European Parliament regulation (Zhang et al., 2023).

Anthocyanins play a pivotal role as pigments, lending the captivating colors to blue honeysuckle juice (Zhao et al., 2020). Given their natural origin, anthocyanins have long attracted significant attention due to their associated health benefits, including antioxidative and anti-inflammatory properties (Zhu et al., 2020). However, anthocyanins are notably fragile and prone to degradation induced by various factors such as pH, temperature, and oxygen exposure (Hernández-Herrero & Frutos, 2015; Navruz, Türkyılmaz, & Özkan, 2016). Consequently, safeguarding anthocyanins from deterioration poses a critical yet formidable challenge. Given their pivotal role in both color and consumer health, protective measures must be implemented during processing and storage to enhance anthocyanin stability (Qian, Liu, Zhao, Cai, & Jing, 2017).

Furthermore, certain phenolics function as effective *co*-pigments and substrates for polymerization, such as flavanols. Both flavan-3-ol monomers and proanthocyanidins can shield the anthocyanin flavylium cation from water's nucleophilic attack through intermolecular co-pigmentation (Cao, Xia, Aniya, Chen, & Jin, 2023; Molaeafard, Jamei, & Poursattar Marjani, 2021), and slow condensation reactions with anthocyanins may also generate polymeric pigments (Roidoung, Dolan, & Siddiq, 2016). Numerous studies have investigated the impact of different co-pigments on anthocyanin coloration in model juice solutions (Hernández-Herrero & Frutos, 2015; Navruz, Türkyılmaz, & Özkan, 2016; Zhang et al., 2019; Roidoung, Dolan, & Siddiq, 2016). Polyphenol co-pigments play a vital role in intensifying and preserving the color of different fruits and vegetables, indicating their ability to form effective co-pigments with anthocyanins (Zhu et al., 2020). However, studies on the mechanism of intermolecular co-pigmentation in model solutions have typically been conducted over short durations (Wang et al., 2008), leaving the long-term effects of co-pigments unresolved. Additionally, the mechanism of polyphenol co-pigmentation on the color protection of blue honeysuckle juice (BHJ) remains undiscovered. Therefore, investigating the influence of polyphenol co-pigments on the color change and development of blue honeysuckle juice is significant.

The objective of this study was to explore the impact of one common phenolic acid (gallic acid) and four common flavonoids (quercetin, rutin, catechin, epigallocatechin gallate (EGCG)) on both the color preservation and antioxidant capacity of BHJ. Fourier transform infrared (FTIR) spectroscopy was employed to analyze the mechanism of *co*-pigmentation between polyphenol co-pigments and anthocyanins. High performance liquid chromatography was used to characterize the components of polyphenol co-pigments and anthocyanins post co-pigmentation. These findings offer a novel approach to enhancing the color stability of blue honeysuckle juice during food processing.

## 2 Materials and methods

### 2.1 Materials

Quercetin was purchased from Macklin Biochemical Co. Ltd. (Shanghai, China). Gallic acid, rutin, catechin, and EGCG were purchased from Yuanye Biological Reagent Co. Ltd. (Shanghai, China). Additionally, 2,2-diphenyl-1-picrylhydrazyl (DPPH), 2,2′-azinobis-(3-ethylbenzthiazoline-6-sulphonate) (ABTS), 2,4,6-tripyridyl-*s*-triazine (TPTZ), and 6-hydroxy-2,5,7,8-tetramethylchroman-2-carboxylic acid (Trolox) were obtained from Sigma-Aldrich corporation (St Louis, MO, USA). HPLC-grade solvents were purchased from Merck Co. Inc. (Darmstadt, German).

The 10-year-old blue honeysuckle cultivar ‘Lanjingling’, a crossbreed between (*L. caerulea* subsp. *altaica* × *L. caerulea* subsp. *kamtschatica*) and L. *caerulea* subsp. *kamtschatica* F_2_, originated from the small berry germplasm resource nursery of Northeast Agricultural Uni*v*ersity, Harbin, Heilongjiang Province, China (126°37′39" E, 45°42’22" N, elevation 127.95 m). In June 2022, ripe blue-black fruits were selected randomly to ensure that they were mature and evenly distributed.

### 2.2 Preparation of BHJ

The blue honeysuckle fruits were mixed with distilled water at a ratio of 1:4 (*w*/*v*) and stirred in a juice blender (Jiuyang, China) for 2 min. The slurry was then centrifuged using a centrifuge (GL-21 M, Iberica, China) at 5000 r/min for 10 min and filtered through a membrane, and the final resulting supernatant constituted the required juice. The juice was then pasteurized at 60 °C for 30 min. The pH of the juice was measured and recorded as 3.42 using a pH meter (METTLER TOLEDO, China), which met the experimental criteria.

### 2.3 Total anthocyanin content (TAC) of BHJ

The TAC in the juice was measured using the pH difference assay with slight modifications (Lee et al., 2005). Potassium chloride (pH = 1.0) and sodium acetate (pH = 4.5) buffers were prepared for diluting the samples. The BHJ samples (20 μL) were mixed with the two buffers (180 μL) in a 96-well plate respectively. The absorbance of the 96-well plate was read at 510 and 700 nm using a microplate reader (BioTek, EPOCH-2, USA). The TAC was quantified using cyanidin-3-glucoside (C3G) equivalent. Its calculation formula is presented in Eq. (1), (2). The TAC in fruits was expressed as milligrams C3G equivalent per liter (DW, dry weight) of the sample. The anthocyanin retention rate was calculated using Eq. (3).

(1) $$A={\left(A_{510\;\mathrm{nm}}-A_{700\;\mathrm{nm}}\right)}_{\mathrm{pH}1.0}-{\left(A_{510\;\mathrm{nm}}-A_{700\;\mathrm{nm}}\right)}_{\mathrm{pH}4.5}$$

(2) $$C3G\;\mathrm{equivalent}\;\left(\mathrm{mg}/L\right)=\frac{A\times \mathrm{MW}\times \mathrm{DF}\times 1000}{\varepsilon \times 1}$$

where A refers to absorbance; MW stands for the molecular weight of C3G (449.2 g/mol); DF represents the dilution factor; ε refers to the molar absorption coefficient of C3G (26900).

(3) $$\mathrm{Anthocyanin retention rate}\;\left(\%\right)=\frac{\mathrm{Total anthocyanin content after storage}}{\mathrm{Initial total anthocyanin content}}\times 100\%$$

### 2.4 Preparation of BHJ *co*-pigmented with polyphenol co-pigments

To enhance the shelf life and color stability of BHJ, we selected five types of polyphenol co-pigments for investigation: gallic acid, quercetin, rutin, catechin, and EGCG. These co-pigments were dissolved in 10 mL of 20 % ethanol in five mass gradients. Subsequently, the co-pigment solutions were added to the juice, resulting in concentration ratios of anthocyanins to polyphenol co-pigments in the juice of 1:0, 1:1, 1:5, 1:10, and 1:20, respectively. The samples were then stored for 24 d at 40 °C in a light incubator, with sampling conducted on days 0, 4, 8, 12, 16, 20, and 24. After sampling, the samples were promptly cooled to 25 °C.

### 2.5 Color determination

The change in color was characterized using the CIE-Lab colorimetric space, employing a Hunter-Lab Color-Flex EZ automatic colorimeter (Hunter-Lab Co.). Color results were represented as L^⁎^ (perception of darkness or lightness), a^⁎^ (level of redness vs. greenness), and b^⁎^ (level of yellowness vs. blueness) (Chen et al., 2022). To visually depict the color changes, samples were photographed under consistent conditions using a mirrorless camera (FUJIFILM X-S10, Japan), and color swatches were generated using Adobe Photoshop CS2 software (Adobe Systems, Inc., San Jose, CA, USA). The change in L^⁎^ (ΔL^⁎^) was computed using Eq. (4). The color contrast between sample pairs was computed as the Euclidean distance between two points within the three-dimensional CIELAB space, employing Eq. (5).

(4) $${\Delta L}^{*}=L^{*}-L_{0}^{*}$$

(5) $${\mathrm{\Delta E}}^{*}=\sqrt{{\left(L_{0}^{*}-L^{*}\right)}^{2}+{\left(a_{0}^{*}-a^{*}\right)}^{2}+{\left(b_{0}^{*}-b^{*}\right)}^{2}}$$

where L_0_^⁎^, a_0_^⁎^, and b_0_^⁎^ are the color parameters of the untreated sample solution (including without co-pigments and storage period).

### 2.6 Determination of antioxidant capacity

#### 2.6.1 DPPH radical scavenging ability

The determination of DPPH followed the Kedare method with modifications (Kedare & Singh, 2011). In general, 60 μM of DPPH solution was first configured in MeOH, after which 5 μL of diluted samples or Trolox and 195 μL of DPPH solution were added to the 96-well plate, incubated at 25 °C in darkness for 2 h. Subsequently, the absorbance was read at 515 nm using a microplate reader. The DPPH inhibition rate of the samples was calculated from the Trolox standard cur*v*e with the formula given in Eq. (6), and the results are expressed as micromoles of Trolox equivalent per gram (DW) of the sample.

(6) $$Y\;\left(\%\right)=\left(1-\frac{{\mathrm{OD}}_{1}}{{\mathrm{OD}}_{0}}\right)\times 100\%$$

where Y stands for the inhibition rate of DPPH of the sample or Trolox. OD_1_ is the absorption value of the sample or Trolox. OD_0_ is expressed as the absorption value of the control.

#### 2.6.2 ABTS radical scavenging ability

Determination of ABTS was referred to a previous assay (Pellegrini, Proteggente, Pannala, Yang, & Rice-Evans, 1999). Initially, the ABTS radical cation solution (ABTS^·+^) was prepared by mixing the ABTS solution (7 mM) with an equal volume (*v*/v) of potassium persulfate solution (2.45 mM), and standing at room temperature in darkness for 12 h. The ABTS^·+^ solution was then diluted with MeOH until the absorbance of solution at 734 nm was measured to be 0.70 ± 0.02. The sample or Trolox (10 μL) and diluted ABTS^·+^ solution (190 μL) were mixed in a 96-well plate and reacted at room temperature in darkness for 6 min, then reading at 734 nm using a microplate reader. The standard cur*v*e was plotted with Trolox and the calculation method utilized Eq. (6). The results are expressed as micromoles of Trolox equivalent per gram (DW) of the sample.

#### 2.6.3 Ferric reducing antioxidant power (FRAP) reduction ability

The FRAP was determined by a previous assay (Zhang, Wong, Wu, Abdul-Karim, & Huang, 2016). Initially, TPTZ (10 mM) mixed with FeCl_3_·6H_2_O (20 mM) and acetate buffer (300 mM, pH = 3.6) to prepare the FRAP reagent with a volume ratio of 1:1:10 (*v*/v/v). The FRAP reagent (150 μL) was heated to 37 °C using a microplate reader and the blank was measured at 593 nm. Then, using MeOH as a control, 10 μL of the samples or FeSO_4_·7H_2_O standards and 30 μL of deionized water were added to a 96-well plate, and the mixture was reacted at 37 °C. The absorbance was measured at 593 nm for 30 min, reading at the 30th min taken as the detection value. Calculated from FeSO_4_·7H_2_O standard curve and the results are expressed as micromoles of Fe^2+^ equivalent per gram (DW) of the sample.

### 2.7 Qualitative and quantitative analysis of anthocyanins using HPLC-ESI-QTOF-MS^2^

High performance liquid chromatography with diode array detection (HPLC-DAD, Agilent, Wilmington, DE, USA) and high-performance liquid chromatography electrospray ionization quadrupole time-of-flight mass spectrometry (HPLC-ESI-QTOF-MS^2^, AB Sciex, CA, USA) were employed, utilizing equipment from Agilent, USA, coupled with a C18 column (Luna 5 μm, 250 mm × 4.6 mm, Phenomenex, CA, USA). For the analysis of polyphenols in BHJ, the column oven temperature was set at 25 °C. The elution system consisted of two solvents: water containing 200 mM formic acid (mobile phase A, pH = 2.6) and acetonitrile containing 50 mM aqueous ammonia acetate (mobile phase B, pH = 3.6). A gradient profile was applied as follows: starting at 14 % B and increasing to 16.5 % B over 0–12.5 min, then further increasing to 25 % B over 12.5–17.5 min, followed by a gradient to 80 % B over 17.5–40 min, a decrease to 50 % B over 40–55 min, and finally decreasing to 14 % B over 55–60 min. The column was subsequently re-equilibrated for 5 min at 5.5 % B, and all runs were conducted at a flow rate of 1 mL/min (Huo et al., 2023; Zhang et al., 2022; Zhang, Chen, et al., 2019). The mass spectrometer was programmed to perform full scanning in positive and negative ion mode at *m/z* 100–2000 (MS^1^) and *m/z* 50–2000 (MS^2^). The capillary voltage for negative ion electrospray ionization (ESI) was set at 4500 *V*, while for positive ion ESI, it was set at 5500 V. The dry temperature was maintained at 550 °C.

### 2.8 Fourier transform infrared (FTIR) spectroscopy analysis

Freeze-drying of BHJ samples to powder in a lyophilizer (LGJ-1A, YATAIKELONG, China) for FTIR spectroscopic analysis. FTIR spectra were acquired using a spectrometer (NICOLET iS50, Thermal Scientific Co., ltd, Shanghai, China) across the wavenumber range from 4000 to 400 cm^−1^ at a resolution of 4 cm^−1^ with a total of 32 scans (Vasincu et al., 2014).

### 2.9 Statistical analysis

The provided data are averages ± standard deviations (in triplicate). Data analysis was performed using SPSS version 27.0, with plot analysis conducted using Origin 2023. One-way analysis of variance (ANOVA) was employed, followed by the minimum significance difference test. Statistical significance was considered for data with *p* < 0.05.

## 3 Results and discussion

### 3.1 Effects of co-pigments on the color of BHJ during storage

**Fig. 1:**
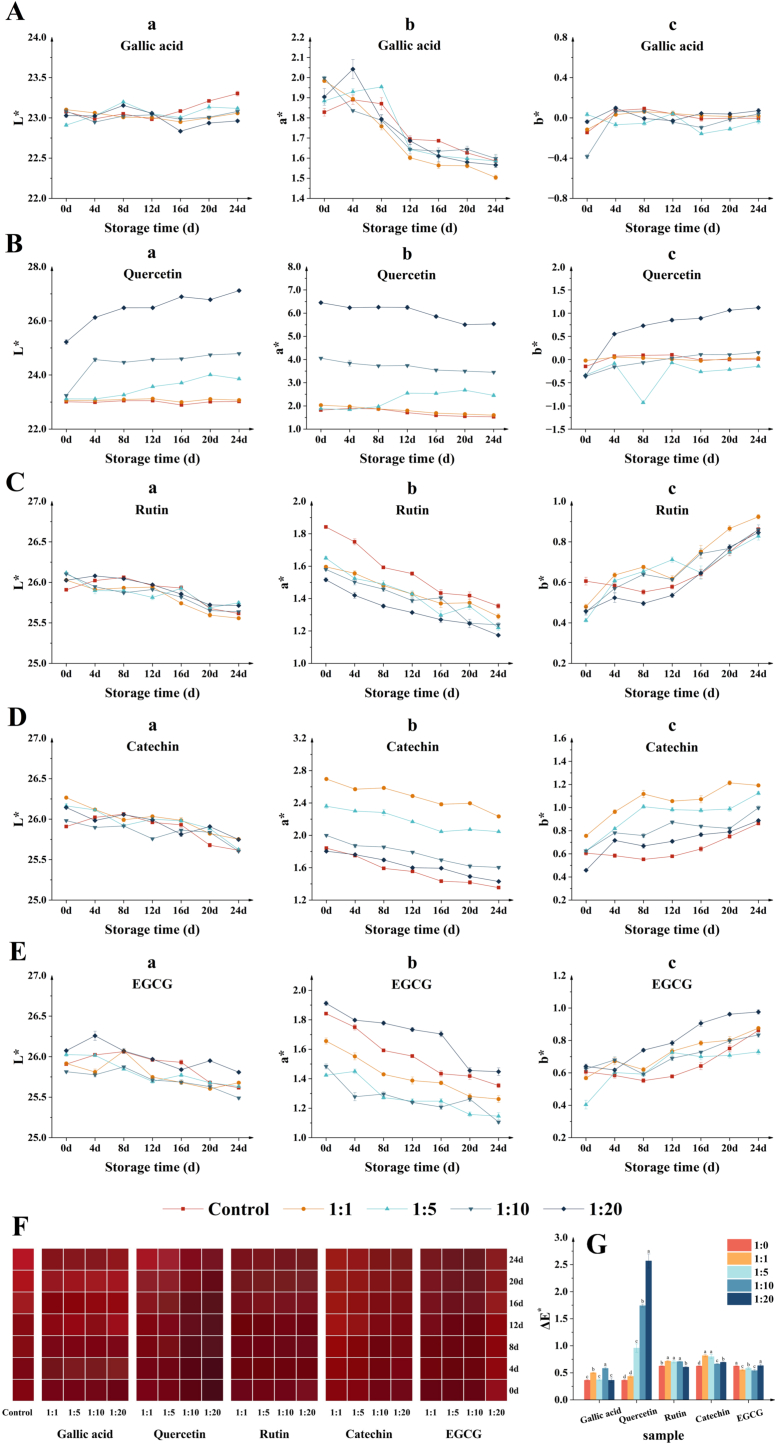
**(A–E)** CIELAB parameters of different BHJ during storage; **(F)** Color swatches. **(G)** Color stability (ΔE^⁎^).

In the presence of varying concentrations of co-pigments, the color stability of BHJ was assessed following a 24 d storage period. The color parameters and swatches of BHJ are shown in Fig. 1. As illustrated in Fig. 1Aa–Ea, the L^⁎^ value of the most BHJ samples exhibited a declining trend during storage. After 24 d, the initial BHJ had an L^⁎^ value of 25.62 ± 0.01, with no significant change observed (ΔL^⁎^ = −0.29, *p* > 0.05). However, the addition of quercetin resulted in a significant increase in the L^⁎^ value of BHJ during storage (*p* < 0.05), with the most notable elevation observed in the 1:20 group, where the L^⁎^ value increased from 25.22 ± 0.09 (0 d) to 27.12 ± 0.04 (24 d), representing a ΔL^⁎^ of 1.90. The unique color-enhancing effect of quercetin may stem from its weak interaction between the hydroxyl group of quercetin and the carbonyl group of anthocyanin (Ertan, Türkyilmaz, & Özkan, 2019), resulting in an increase in the L^⁎^ value in BHJ. The ΔL^⁎^ of BHJ containing other polyphenol co-pigments was significantly lower compared to that of the original BHJ except for quercetin (*p* < 0.05). Among these, the L^⁎^ of the catechins (1:5) group exhibited the most pronounced decrease with a ΔL^⁎^ of −0.54, followed by the rutin (1:1) group with a ΔL^⁎^ of −0.47. Lower L^⁎^ values in BHJ supplemented with co-pigments were associated with a redder color tone, possibly due to their blocking effect on anthocyanin degradation, thus preventing color fading.

As shown in Fig. 1Ab–Eb, overall, following a 24 d storage, the a^⁎^ value of BHJ showed a decreasing trend, accompanied by a gradual fading of the red color. A significant difference in a^⁎^ due to co-pigmentation was observed compared to the control group (p < 0.05). Notably, the BHJ containing quercetin (1:20) demonstrated the highest redness after 24 d of storage, with an a^⁎^ value of 5.54 ± 0.03, indicating quercetin's superior color enhancement effect. This was followed by catechin (1:1), with an a^⁎^ value of 2.23 ± 0.02. However, the impact of gallic acid on the a^⁎^ value of BHJ was not significant (*p* > 0.05), failing to meet the expectations of color protection. Interestingly, lower concentrations (1:1) of catechins and rutin exhibited a more pronounced red effect, while the a^⁎^ value increased with quercetin concentration. This phenomenon may be attributed to the color of the co-pigments themselves. Both catechin and rutin are pale green powders, and high concentrations of them prompt the transition of the BHJ to greenness. Whereas quercetin molecules have a different three-dimensional structure, electron-absorbing capacity and variation in molecular flatness than catechin and rutin, the presence of hydroxyl groups that bind anthocyanins with a higher strength leads to discrepancies in the final co-pigmentation effect (Cheng et al., 2023).

In terms of b^⁎^, as shown in Fig. 1Ac–Ec, the b^⁎^ value of all samples significantly increased after 24 d of storage (*p* < 0.05), indicating a shift in the color of BHJ from redness to yellowness, irrespective of the co-pigment used. The b^⁎^ value of BHJ containing catechin (1:1) was notably higher than that of other treatments (1.19 ± 0.01), resulting in a more yellowish hue. This was followed by quercetin (1:20), with a b^⁎^ value of 1.12 ± 0.03. High concentrations (1:20) of quercetin and EGCG significantly affected the b^⁎^ value of BHJ (1.12 ± 0.03, 0.98 ± 0.01), while low concentrations (1:1) of rutin and catechin also had significant effects on the b^⁎^ value of BHJ (0.92 ± 0.01, 1.19 ± 0.01). Gallic acid, however, did not exert a significant effect on the b^⁎^ value of BHJ (*p* > 0.05).

The color swatches and ΔE^⁎^ values of BHJ with varying concentrations of co-pigments are illustrated in Fig. 1F and G. A lower ΔE^⁎^ value indicates better color stability of BHJ during storage. As depicted in Fig. 1F, color fading was evident in all samples after 24 d of storage, consistent with the gradual decrease in a^⁎^ value. For the control group, the original BHJ retained its bright red color for 12 d during storage; however, with prolonged storage, significant color fading occurred (ΔE^⁎^ = 0.62 ± 0.01, *p* < 0.05). In comparison to the control group, a high concentration of quercetin (1:20) noticeably enhanced the color depth of BHJ. However, its stability decreased as the concentration deepened (ΔE^⁎^ = 2.56 ± 0.13). This trend was also observed with EGCG, which intensified the color of BHJ at concentrations of 1:1, 1:5, and 1:10. Conversely, high concentrations (1:20) of EGCG resulted in color damage to BHJ (ΔE^⁎^ = 0.63 ± 0.02). Higher concentrations of catechin (1:10) and lower concentrations of rutin (1:1) significantly intensified the color of BHJ with better color stability (ΔE^⁎^ = 0.66 ± 0.01, 0.55 ± 0.03). In the presence of gallic acid, the color swatches of BHJ did not exhibit significant changes (ΔE^⁎^ = 0.36 ± 0.04 for 1:20, *p* > 0.05). In summary, the order of color protection effects of the five co-pigments on BHJ was observed as follows: quercetin > EGCG > rutin > catechin > gallic acid. Quercetin and EGCG molecules are typically conjugated planar molecules devoid of glycosidic elements, forming strongly stack-stabilizing complexes with anthocyanins. Rutin molecules, with a long-branched chain containing a disaccharide glycoside in the C ring, readily form robust hydrogen bonds with molecules having numerous hydroxyl groups. Catechin molecules, due to the torsion of the B ring, exhibit poor conjugation. Gallic acid molecules, while modifying anthocyanins to a lesser extent compared to flavonoids, results in a significant discrepancy in their co-chromatic effect (Zhang et al., 2015).

### 3.2 Effect of co-pigments on TAC in BHJ during storage

**Fig. 2:**
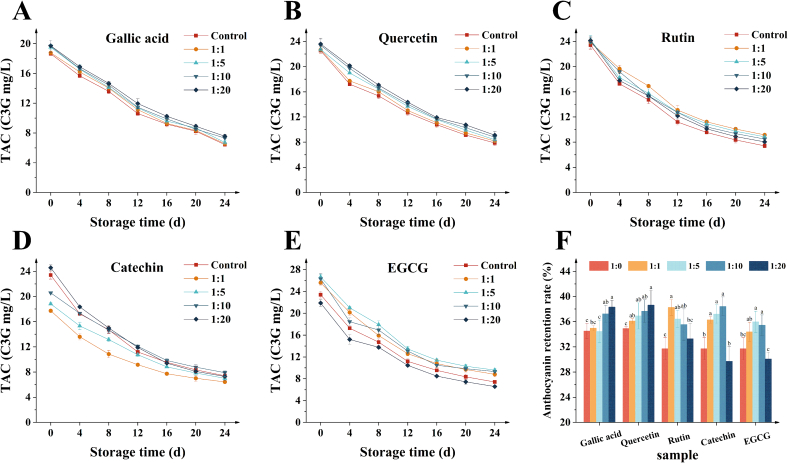
**(A–E)** TAC of different BHJ during storage; **(F)** Anthocyanin retention rate.

The TAC and anthocyanin retention rates of BHJ with different concentrations of co-pigments over a 24 d storage is shown in Fig. 2A–F, respectively. Throughout the 24 d of storage, there was a significant decreasing trend in TAC across all BHJ samples (*p* < 0.05). Notably, compared to the control group, BHJ supplemented with EGCG (1:5) exhibited the highest TAC (9.59 ± 0.33 mg/L) after 24 d, with an anthocyanin retention rate of 35.98 ± 1.69 %. Following closely was the quercetin (1:20) group, with a TAC of 9.21 ± 0.55 mg/L and anthocyanin retention rate of 38.62 ± 2.32 %. Interestingly, there were discrepancies in the optimal concentration of flavonoids for anthocyanin protection. As illustrated in Fig. 2B and D, the highest anthocyanin retention rates were observed with higher concentrations of quercetin (1:20) and catechin (1:10) added to BHJ, yielding rates of 38.62 ± 2.32 % and 38.43 ± 1.50 %, respectively. Conversely, Fig. 2C and E demonstrated that lower concentrations of rutin (1:1) and EGCG (1:5) exhibited the best protective capacity against BHJ anthocyanins within the same group, with retention rates of 38.28 ± 1.24 % and 35.98 ± 1.69 %, respectively. This variation in protection effectiveness could be attributed to the ability of anthocyanins and flavonoids to form complexes with face-to-face stacked structural arrangements, resulting in stronger bonds (Huang et al., 2023). Notably, this conformational capacity is related to the spatial structure in which the co-pigments and anthocyanins are situated. EGCG forms highly compact complexes with anthocyanins due to its nearly parallel molecular planes, resulting in the strongest interaction. Quercetin also forms a parallel compact structure but exhibits slightly lower interaction strength compared to EGCG. Rutin, with a larger molecular structure, forms complexes less efficiently but still maintain strong interactions through hydrogen bonding with its hydroxyl groups. Conversely, the anthocyanin-catechin complex, with its non-planar structure, displays the weakest interaction (Cao et al., 2023). These findings highlight the significant influence of co-pigment spatial structure on co-pigmentation performance, impacting the nature of interactions with anthocyanins.

Fig. 2A illustrates that phenolic acids were less effective than flavonoids in influencing the TAC of BHJ. Nonetheless, adding a higher mass ratio of gallic acid (1:20) significantly improved the TAC and anthocyanin retention rate after 24 d of storage (*p* < 0.05), resulting in a TAC of 7.56 ± 0.25 mg/L and an anthocyanin retention rate of 38.32 ± 1.08 %. This improvement is attributed to the antioxidant properties of phenolic acids, which protect anthocyanins from oxidation (Zhang, Chen, et al., 2019; Zhang, Wang, et al., 2019). Phenolic acids act as co-pigments by forming noncovalent bonds with anthocyanins to create anthocyanin-cofactor complexes that resist nonoxidative degradation, such as hydration and further molecular breakdown (Zhang et al., 2018). In contrast, flavonoids not only serve as effective co-pigments, engaging in intermolecular interactions with anthocyanins to form stable complexes, but also drive the hydration balance reaction of anthocyanins toward the flavylium cation form, thereby providing greater protective effects compared to phenolic acids (Kanha, Surawang, Pitchakarn, Regenstein, & Laokuldilok, 2019). To summarize, the protective efficacy of polyphenol co-pigments on anthocyanins ranks in the order: EGCG > quercetin > rutin > catechin > gallic acid.

### 3.3 Effect of co-pigments on antioxidant capacity of BHJ during storage

**Fig. 3:**
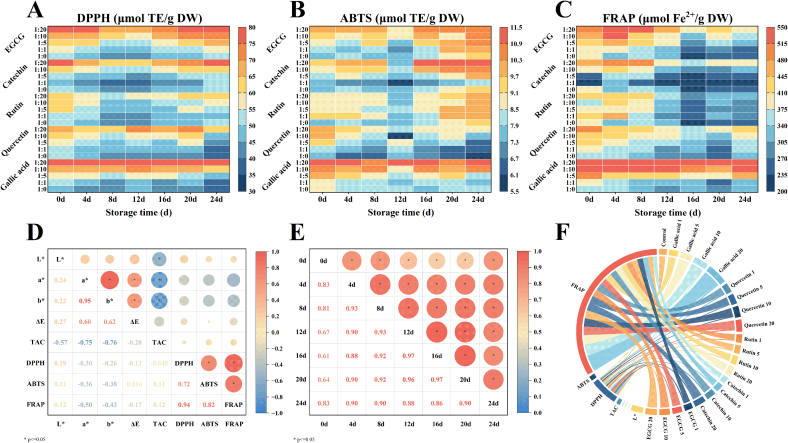
**(A–C)** DPPH, ABTS, and FRAP antioxidant capacities; **(D)** Pearson's correlation coefficient between L^⁎^, a^⁎^, b^⁎^, ΔE^⁎^, TAC, DPPH, ABTS, and FRAP of BHJ; Asterisks (^⁎^) indicates significant correlation at *p* ≤ 0.05; **(E)** Pearson's correlation coefficient between storage periods (0 d, 4 d, 8 d, 12 d, 16 d, 20 d and 24 d) of BHJ; **(F)** Correlation analysis.

The antioxidant capacity of BHJ was assessed using ABTS, DPPH, and FRAP methods, which measure the ability of antioxidants to neutralize free radicals through electron transfer and hydrogen atom transfer mechanisms, either independently or concurrently (Loizzo et al., 2009). Fig. 3A–C demonstrates a significant enhancement in BHJ's antioxidant capacity with increasing concentrations of co-pigments (*p* < 0.05). However, there were slight variations in the effectiveness of polyphenol co-pigments in boosting BHJ's antioxidant capacity, likely due to their inherent strong antioxidant properties and their interaction with anthocyanins in BHJ.

After 24 d of storage, a notable decrease in antioxidant capacity was observed in most BHJ samples compared to the non-stored control (p < 0.05). Notably, the addition of a high concentration of gallic acid (1:20) demonstrated the most significant enhancement in DPPH and FRAP antioxidant capacity of BHJ, with values significantly higher than other sample groups (p < 0.05). These values were 2.11 and 2.41 times higher than those of the control group (77.35 ± 0.69 μmol TE/g DW, 790.76 ± 9.89 μmol Fe^2+^/g DW). Similarly, a high concentration of quercetin (1:20) notably increased the ABTS antioxidant capacity of BHJ by 1.49 times compared to the control (8.74 ± 0.22 μmol TE/g DW). In contrast, rutin at a high concentration (1:20) exhibited the least enhancement effect, with DPPH, ABTS, and FRAP antioxidant capacities after 24 of storage only 1.25, 1.09, and 1.27 times higher than those of the control group (62.55 ± 0.46 μmol TE/g DW, 10.11 ± 0.06 μmol TE/g DW, 307.43 ± 0.67 μmol Fe^2+^/g DW), respectively.

In summary, the ranking of polyphenol co-pigments based on their effect on BHJ's antioxidant capacity was as follows: gallic acid > quercetin > EGCG > catechin > rutin for DPPH; quercetin > gallic acid > catechin > EGCG > rutin for ABTS; and gallic acid > EGCG > catechin > quercetin > rutin for FRAP. The configuration of polyphenol co-pigments, particularly the quantity and position of hydroxyl groups, significantly influences their antioxidant capacity of BHJ. Also, the synergistic interactions of polyphenol co-pigments with anthocyanins in BHJ, facilitating extraction and availability of these compounds. The formation of complexes between the two enhances anthocyanin retention, also contributing significantly to their superior antioxidant capacity (Klisurova et al., 2019).

### 3.4 Correlation analysis of measurement indicators and storage time

Color parameters, TAC, and antioxidant capacity were evaluated using Pearson correlation, which employs direct linear regression analysis (Xiao et al., 2023). Regarding measurements (Fig. 3D and F), the strongest correlation was observed between the a^⁎^ and b^⁎^ color parameters (*r* = 0.95). TAC exhibited negative correlations with color parameters (L^⁎^, a^⁎^, b^⁎^) (*r* = −0.57, −0.75, −0.76), suggesting that anthocyanin content significantly influenced the color characteristics of BHJ. Additionally, a notable positive correlation was found among these antioxidant capacity (DPPH, ABTS, and FRAP), indicating consistent and ordered effects of polyphenol co-pigments on BHJ's antioxidant capacity. However, no significant correlation was observed between antioxidant capacity (DPPH, ABTS, and FRAP) and color parameters (L^⁎^, a^⁎^, b^⁎^, ΔE^⁎^) or TAC (−0.5 < *r* < 0.5). In terms of storage time (Fig. 3E), correlation coefficients between each time point exceeded 0.8, except for day 0, indicating a significant positive correlation between storage duration and observed physiological changes.

### 3.5 FTIR analysis for molecular structure investigation of co-pigmented BHJ

**Fig. 4:**
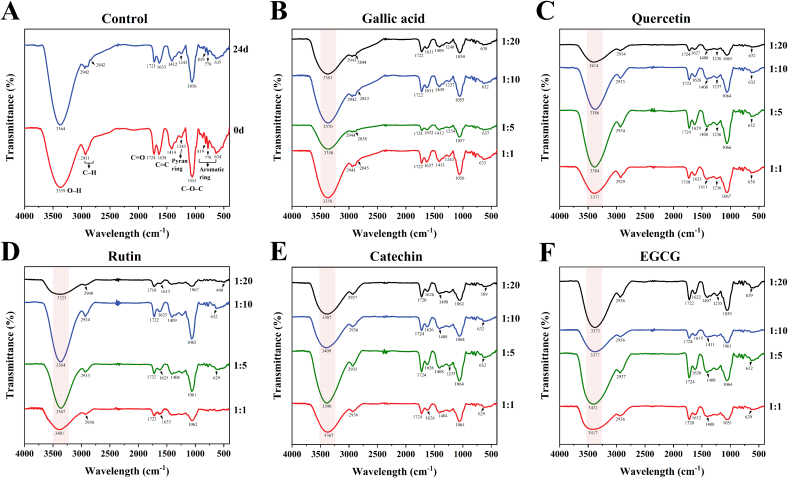
FTIR spectra of BHJ. **(A)** Control; **(B–F)** Treatment after 24 d of storage.

To further understand the protective mechanism of polyphenol co-pigments on the stability of BHJ, the functional groups in BHJ were identified and confirmed using FTIR spectroscopy, with analysis results presented in Fig. 4A–F. In Fig. 4A, the obtained spectrum revealed significant features: the broad band at 3359 cm^−1^ corresponding to O—H stretching vibrations (Liu et al., 2020), the absorption band at 2931 cm^−1^ indicative of asymmetrical stretched C—H groups, and the peak at 1724 cm^−1^ associated with carbonyl groups. The absorption at 1055 cm^−1^ represented C—O—C flexural vibrations, likely due to the presence of carbohydrates. The band at 1638 cm^−1^ suggested C=C shear vibrations, while the peak at 1243 cm^−1^ indicated pyran ring stretching, characteristic of flavonoids. Additionally, several absorption bands around 819 to 634 cm^−1^ indicated aromatic ring vibrations (Molaeafard et al., 2021). Comparing with the control group, the BHJ spectrum after 24 d of storage exhibited a new peak at 2842 cm^−1^, indicating the appearance of a new symmetrically stretched C—H group in anthocyanins. In addition, the decreased signals at 1412 to 1243 cm^−1^ indicated weakened pyran ring stretching and reduced flavonoid content. This may be due to degradation of anthocyanins after 24 d of storage. Similarly, the absorption signal of bands around 819–779 cm^−1^ decreased, suggesting weakened aromatic ring vibrations and reduced phenolic content.

In Fig. 4B–F, the O—H stretching vibration characteristic peak of BHJ shifted significantly after adding polyphenol co-pigments compared to the control group. Among them, the EGCG group exhibited the largest shift, with the maximum peak shifting from 3364 cm^−1^ (control) to 3432 cm^−1^ (1:1). Quercetin followed with a peak shift to 3414 cm^−1^ (1:20), while gallic acid showed the smallest shift, reaching only 3381 cm^−1^ (1:20). This shift can be attributed to the formation of noncovalent interactions, i.e. intermolecular hydrogen bonds, between the anthocyanin and polyphenol co-pigments in the BHJ (Zhao, He, Zhang, Shi, & Duan, 2022). In addition, the O—H group vibration peak offset of gallic acid and quercetin groups increased with higher concentrations of added co-pigments, while the concentration of rutin, catechin and EGCG groups shown that the maximum deviation of 1:1, 1:10 and 1:5, respectively, aligning with anthocyanin retention rates. In Fig. 4C, the spectral intensity of the quercetin (1:20) group significantly decreased from 1723 cm^−1^ to 632 cm^−1^, suggesting interaction between hydroxyl groups of BHJ anthocyanins and quercetin's carboxyl group or oxygen atom. Fig. 4D shows that a high concentration of rutin (1:20) led to smaller wavelengths of O—H group vibrational peaks. This may be due to increased hydrogen bonding and larger π-π stacking interaction regions within the complex formed by rutin (Huang et al., 2023). However, the nonplanar surface of rutin created an intermolecular barrier, causing a shift in O—H group vibrational peaks. In Fig. 4F, the spectrum peak at 1235 cm^−1^ significantly intensified with the addition of a high concentration (1:20) of EGCG, attributed to the pyran ring stretching vibration of EGCG itself (Chen et al., 2022).

In summary, the FTIR spectra results indicate that conformational rearrangements of the complex molecules formed between anthocyanins and polyphenol co-pigments in BHJ generate new binding forces, where hydrogen bonding achieves optimal protection of the anthocyanins and is a significant driver of color protection in BHJ.

### 3.6 Characterization and quantification of anthocyanins in BHJ

**Fig. 5:**
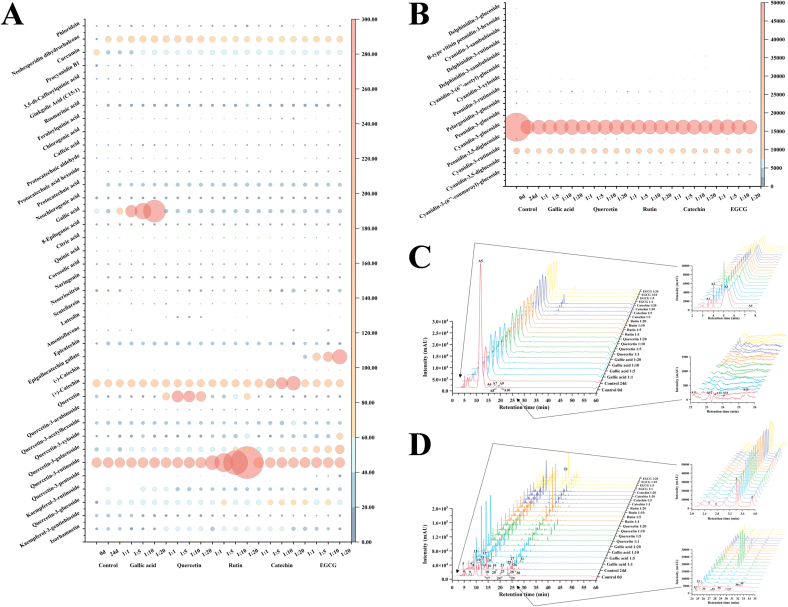
**(A)** Non-anthocyanin polyphenol contents (mg/100 g); **(B)** Anthocyanin contents (mg/100 g); **(C)** HPLC chromatogram (UV 520 nm) of anthocyanins and **(D)** HPLC chromatogram (UV 280 nm) of non-anthocyanin polyphenols from the BHJ.

The anthocyanins in BHJ were identified and quantified using HPLC-ESI-QTOF-MS^2^ (refer to Fig. 5C). The corresponding mass spectrometry and quantitative data can be found in Table S1, Table 1, Fig. S1 and Fig. 5B.

**Table 1:**
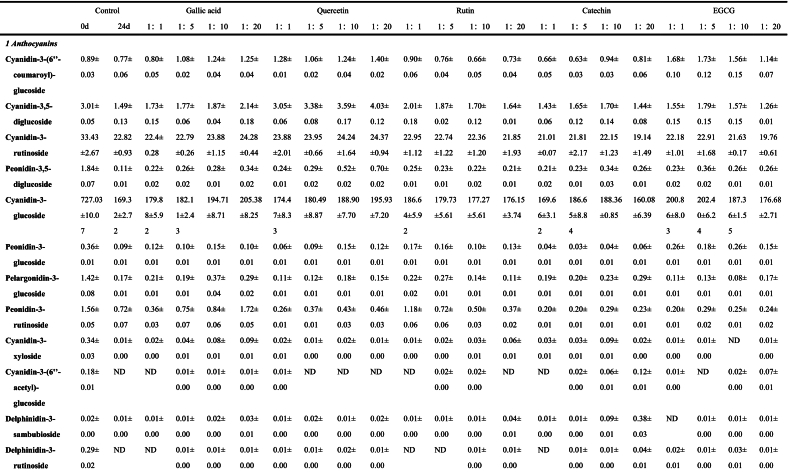

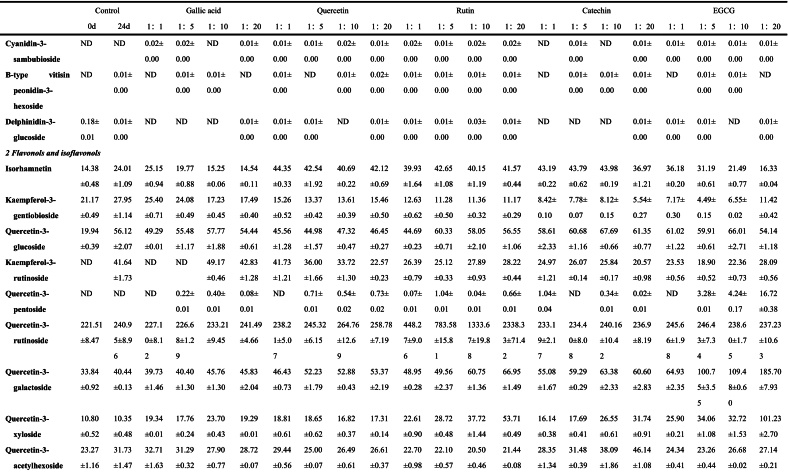

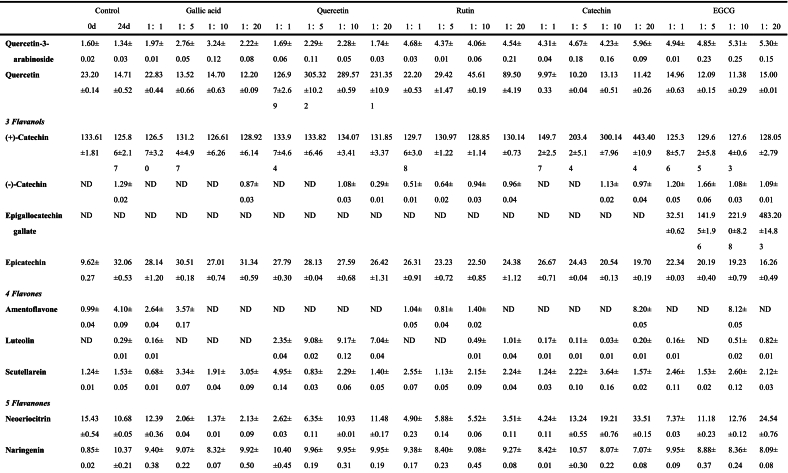

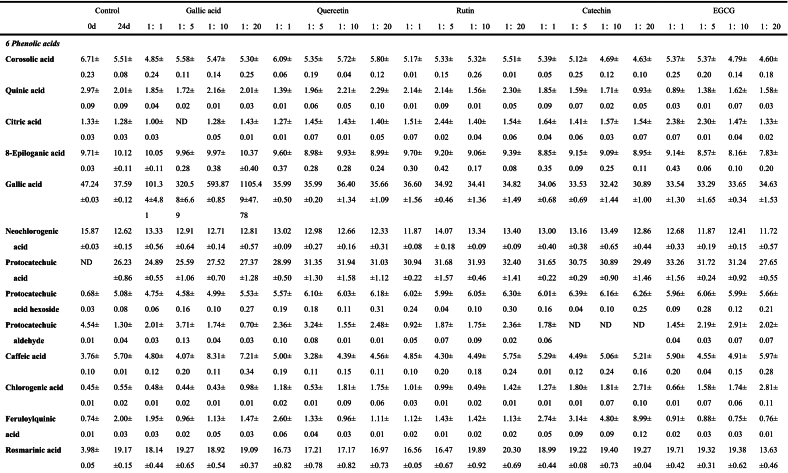

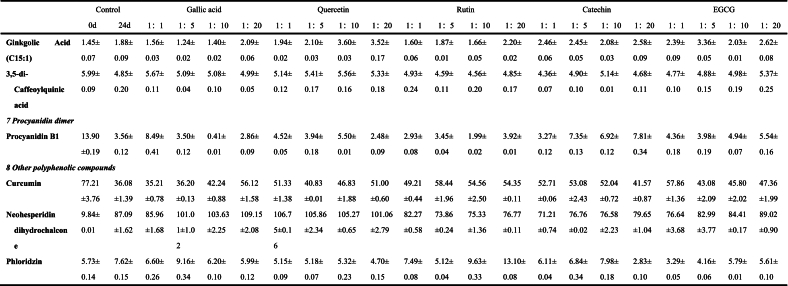
Characterization and quantification of phenolic compounds of BHI (mg/100 g).

Data are expressed as mean ± standard deviation (*n* = 3). ND = Not detected.

Peaks A1 (*m/z* 595), A2 (*m/z* 611), A3 (*m/z* 595), A5 (*m/z* 449), A9 (*m/z* 419), A10 (*m/z* 491), and A13 (*m/z* 581) yielded identical fragment *m/z* 287, which corresponds to cyanidin aglycon (Chaovanalikit, Thompson, & Wrolstad, 2004) and is derived from the loss of a glucoside moiety *m/z* 162, coumaroylglucoside molecule [M-308]^+^, diglucoside molecule [M-324]^+^, rutinoside molecule [M-308]^+^, xyloside molecule [M-132]^+^, acetylglucoside molecule [M-204]^+^, and sambubioside molecule [M-294]^+^, respectively. Therefore, the peaks A1, A2, A3, A5, A9, A10, and A13 were tentatively identified as cyanidin-3-(6`’-coumaroyl)-glucoside, cyanidin-3,5-diglucoside, cyanidin-3-rutinoside, cyanidin-3-glucoside, cyanidin-3-xyloside, cyanidin-3-(6`’-acetyl)-glucoside and cyanidin-3-sambubioside, respectively.

Similarly, peaks A4 (*m/z* 625), A6 (*m/z* 463), and A8 (*m/z* 609) exhibited the same fragment *m/z* 301, which equates with peonidin aglycon (Oszmianski, Wojdylo, & Lachowicz, 2016) and indicate that it eliminated the diglucoside molecule [M-324]^+^, glucoside molecule [M-162]^+^, and rutinoside molecule [M-308]^+^, respectively. Therefore, peaks A4, A6, and A8 were tentatively identified as peonidin-3,5-diglucoside, peonidin-3-glucoside, and peonidin-3-rutinoside, respectively. Peaks A11 (*m/z* 597), A12 (*m/z* 611), and A15 (*m/z* 465) also yielded the same fragment *m/z* 303, which corresponds to delphinidin aglycon (Chaovanalikit et al., 2004) and were derived from the loss of the sambubioside molecule [M-294]^+^, rutinoside molecule [M-308]^+^, and glucoside molecule [M-162]^+^, respectively. Peaks A11, A12, and A15 were tentatively characterized as delphinidin-3-sambubioside, delphinidin-3-rutinoside, and delphinidin-3-glucoside, respectively.

Peak A7 was identified as pelargonidin-3-glucoside, confirmed by the molecular ion *m/z* 433 [M]^+^ and fragmentation ions of *m/z* 162 and *m/z* 271, representing the loss of a glucose and pelargonidin residues. Peak A14 was tentatively assigned as B-type vitisin peonidin-3-hexoside (Oszmianski et al., 2016), a pyranoanthocyanin, based on the molecular ion *m/z* 487 [M]^+^, along with fragment ions of *m/z* 325 [M-162]^+^ indicative of glucose loss and *m/z* 301 [peonidin]^+^.

Among the 15 characterized anthocyanins in BHJ (Table 1 and Fig. 5B), cyanidin-3-glucoside, cyanidin-3-rutinoside, and cyanidin-3,5-diglucoside were predominant. However, after 24 d of storage, most anthocyanin contents in BHJ significantly decreased (*p* < 0.05), highlighting the impact of storage on anthocyanin stability. The cyanidin-3-glucoside content decreased from 727.03 ± 10.07 to 160.08 ± 6.39 mg/100 g DW, while the cyanidin-3-rutinoside content decreased from 33.43 ± 2.67 to 19.14 ± 1.49 mg/100 g DW. BHJ with polyphenol co-pigmentation exhibited significantly higher anthocyanin content compared to the control group (p < 0.05). Using cyanidin-3-glucoside as a reference, the gallic acid (1:20) group had the highest cyanidin-3-glucoside content among all treatments at 205.38 ± 8.25 mg/100 g DW, followed by the EGCG (1:5) and (1:1) groups with cyanidin-3-glucoside contents of 202.40 ± 6.24 and 200.86 ± 8.03 mg/100 g DW, respectively. The lowest cyanidin-3-glucoside content was found in the catechin (1:20) group at 160.08 ± 6.39 mg/100 g DW. The quercetin (1:20), catechin (1:10), and rutin (1:1) groups showed the highest cyanidin-3-glucoside content within their respective co-pigmentation ranges, with values of 195.93 ± 7.20, 188.36 ± 0.85, and 186.64 ± 5.92 mg/100 g DW, respectively.

In summary, polyphenol co-pigments exhibit excellent protective ability against anthocyanins in BHJ. This protective effect can be attributed to the stabilization of the purple dehydrated base in the BHJ, preventing its conversion to the colorless chalcone pseudo-base structure and thus enhancing the color-presenting effect of the anthocyanin (Asen, Stewart, & Norris, 1975). These co-pigments form stable complexes with anthocyanins such as cyanidin-3-glucoside through hydrogen bonding and intermolecular van der Waals forces. These complexes protect the flavylium cation of anthocyanins, thereby effectively preventing their degradation (Kanha et al., 2019).

### 3.7 Characterization and quantification of non-anthocyanin polyphenols in BHJ

Non-anthocyanin polyphenols in BHJ were characterized and quantified, and the HPLC chromatogram was shown in Fig. 5D. The corresponding MS data and quantitative results are summarized in Table S1, Table 1, Fig. S1 and Fig. 5A.

#### 3.7.1 Flavonols and isoflavonols

Peaks 11,19, 28, 30, 31, 33, and 35 yielded the same fragment *m/z* 301, indicative of a quercetin moiety resulting from the loss of different glycoside fractions (162 Da for glucoside, 132 Da for pentoside, 308 Da for rutinoside, 162 Da for galactoside, 132 Da for xyloside, 204 Da for acetohexoside, and 132 Da for arabinoside (Palíková et al., 2008). Therefore, peaks 11, 19, 28, 30, 31, 33, and 35 were tentatively characterized as quercetin-3-glucoside, quercetin-3-pentoside, quercetin-3-rutinoside, quercetin-3-galactoside, quercetin-3-xyloside, quercetin-3-acetylhexoside, and quercetin-3-arabinoside, respectively. Peak 37 yielded an *m/z* of 301 [M-H]^−^, corresponding to a quercetin fragment and tentatively designated as quercetin. Peaks 7 and 13 yielded a common fragment at *m/z* 285, typical of kaempferol fragments but with different glycosyl structures, specifically gentiobioside (324 Da) and rutinoside (308 Da), respectively. Therefore, peaks 7 and 13 were tentatively identified as kaempferol-3-gentiobioside and kaempferol-3-rutinoside (Zhang et al., 2023). The parent ion of peak 6 was *m/z* 315 [M-H]^−^, corresponding to isorhamnetin.

#### 3.7.2 Flavanols

Peaks 15, 16, and 23 yielded the anion *m/z* 289 [M-H]^−^, characteristic of catechins or epicatechins (Xiao et al., 2023). Peaks 15 and 16 share a common fragment at *m/z* 289, and these substances are presumed to be (+)-catechin and (−)-catechin, respectively. The fragment observed at *m/z* 178 for peak 23, resulting from the removal of water molecules, suggests it could be epicatechin. Peak 22 exhibited an anion at *m/z* 458 [M-H]^−^ and a fragmentation ion at *m/z* 305, typical of epigallocatechin, with a gallate group removed (153 Da). Therefore, peak 22 was tentatively identified as EGCG.

#### 3.7.3 Flavones

Peaks 36 and 39 exhibited MS at *m/z* 285 [M-H]^−^, with the molecular formula C_15_H_10_O_6_ for both isomers. Based on MS/MS results, peaks 36 and 39 were identified as luteolin and scutellarein, respectively. Tandem mass spectrometry indicated that the deprotonated ion of peak 8 was at *m/z* 537 [M-H]^−^, with a fragment ion at *m/z* 375, suggesting that peak 8 might be amentoflavone (Oszmianski et al., 2016).

#### 3.7.4 Flavanones

Peak 25 is a flavanone that produces a characteristic anionic fragment at *m/z* 595 [M-H]^−^ and an ionic fragment at *m/z* 301. Therefore, it is hypothesized to be neoeriocitrin. Peak 38 displays a parent ion at *m/z* 271 [M-H]^−^ and a fragment at *m/z* 119, which is typical of naringenin and its derivatives (Palíková et al., 2008). Consequently, peak 38 was tentatively identified as naringenin.

#### 3.7.5 Phenolic acids

Peaks 10, 21, 24, and 32 yielded the same fragment *m/z* 191, indicative of the quinic acid moiety. Among these, peaks 10 and 21 shared an anion at *m/z* 353 [M-H]^−^ (Oszmianski et al., 2016). Peaks 24 and 32 displayed a feruloyl fragment (176 Da) and a 3,5-di-caffeoyl fragment (324 Da), respectively. Hence, peaks 10, 21, 24, and 32 were tentatively identified as neochlorogenic acid, chlorogenic acid, feruloylquinic acid, and 3,5-di-caffeoylquinic acid, respectively. Peaks 4 exhibited an anion of *m/z* 375 [M-H]^−^ and a fragment ion of *m/z* 213. Therefore, peaks 4 was tentatively assigned as 8-epimachanic acid. Peaks 2 and 3 produced an anion at *m/z* 191 [M-H]^−^, corresponding to chemical formulas C_7_H_12_O_6_ and C_6_H_8_O_7_, respectively. Thus, peaks 2 and 3 were preliminarily identified as quinic acid and citric acid. Peak 12 was tentatively identified as protocatechuic acid, evidenced by its ability to yield an anion at *m/z* 153 [M-H]^−^ and a fragment ion at *m/z* 108. The fragment ion produced by peak 14 (*m/z* 153 [M-H-162]^−^) may be associated with the loss of hexoside (162 Da). Thus, peak 14 was tentatively identified as protocatechuic acid hexoside (Zhang et al., 2023). Peak 17, with an MS at *m/z* 137 [M-H]^−^ and MS/MS at *m/z* 108 and 137, was identified as protocatechuic aldehyde (Fernandes, Sousa, Mateus, Cabral, & De Freitas, 2011). Peaks 5 and 20 exhibited anions at *m/z* 169 [M-H]^−^ and *m/z* 179 [M-H]^−^, with fragment ions at *m/z* 123 and *m/z* 89, respectively, characteristic of gallic acid and caffeic acid (Caprioli et al., 2016). Peak 27, with an anion at *m/z* 359 [M-H]^−^ and fragment ions at *m/z* 197, was tentatively identified as rosmarinic acid.

#### 3.7.6 Procyanidin dimer

Peak 18 (*m/z* 577 [M-H]^−^) experienced retro-Diels-Alder fission of the heterocyclic ring and cleavage of the interflavanyl bond, leading to the formation of a fragmentation ion at *m/z* 289 [M-H-289]^−^. In addition, fragmentation of peak 18 was shown by breakage of the 4 → 8 and 4 → 6 attachment sites, resulting in the generation of monomers and characteristic residues containing part of the procyanidin (*m/z* 407 [M-H-170]^−^) (Zhang et al., 2022). Therefore, peak 18 was tentatively identified as procyanidin B1.

#### 3.7.7 Quantification of non-anthocyanin polyphenols in BHJ

The quantification results of non-anthocyanin polyphenols in the juice are presented in Table 1 and Fig. 5A. A total of 39 non-anthocyanin polyphenols was identified in the BHJ, including 11 flavonols and isoflavonols, 4 flavanols, 3 flavones, 2 flavanones, 15 phenolic acids, 1 procyanidin dimer, and 3 other polyphenols. This number is fewer compared to the crude extract of polyphenols from blue honeysuckle (Zhang et al., 2023), possibly due to the loss of polyphenols during juice processing. Flavonols and isoflavones were the predominant non-anthocyanin polyphenols in BHJ, ranging from 369.70 ± 13.59 to 2712.65 ± 80.12 mg/100 g DW. Phenolic acids were the most diverse, with contents ranging from 105.42 ± 0.84 to 1208.19 ± 51.54 mg/100 g DW, exhibiting potential anti-inflammatory, antibacterial, and antioxidant bioactivities (Fernandez-Panchon, Villano, Troncoso, & Garcia-Parrilla, 2008).

We found a significant increase in the non-anthocyanin polyphenol content of BHJ after 24 d of storage compared to the control, attributed to the degradation and metabolism of anthocyanins into non-anthocyanin polyphenols in BHJ (Oszmianski et al., 2016). Quercetin, primarily present in glycoside form, emerged as the most abundant flavonol in BHJ. Apart from rutin and quercetin, quercetin-3-galactoside (ranging from 33.84 ± 0.92 to 185.70 ± 7.93 mg/100 g DW) was the predominant flavonol, followed by quercetin-3-glucoside (19.94 ± 0.39 to 67.69 ± 0.66 mg/100 g DW) and kaempferol-3-rutinoside (18.90 ± 0.52 to 49.17 ± 0.46 mg/100 g DW). EGCG had a profound impact on flavonol content in BHJ. Addition of EGCG significantly increased the relative content of quercetin-3-galactoside, quercetin-3-xyloside, and quercetin-3-pentoside in BHJ, reaching levels of 185.70 ± 7.93, 101.23 ± 2.70, and 16.72 ± 0.38 mg/100 g DW, respectively, in the EGCG 1:20 sample group. Concurrently, EGCG down-regulated the content of isorhamnetin, reaching a minimum relative content of 16.33 ± 0.04 mg/100 g DW (EGCG 1:20). The enhancement of quercetin-3-galactoside (66.95 ± 1.49 mg/100 g DW in 1:20) and quercetin-3-xyloside (53.71 ± 0.49 mg/100 g DW in 1:20) by rutin was particularly notable. This effect may be attributed to the breakdown of quercetin glycosides in rutin during storage, leading to the formation of new monomeric substances with free galactosides and xylosides (Fernandez-Panchon et al., 2008). The range of relative content of phenolic acids in BHJ after 24 d of light storage was 129.89 ± 4.61 to 145.07 ± 4.67 mg/100 g DW, excluding the gallic acid and control groups. Rutin significantly up-regulated the relative content of phenolic acids, whereas EGCG acted as a significant down-regulator. For instance, the rosemarinic acid content in BHJ with 1:1 rutin and EGCG was 16.56 ± 0.05 and 19.71 ± 0.42 mg/100 g DW, respectively, while with 1:20, it was 19.89 ± 0.92 and 13.63 ± 0.46 mg/100 g DW, respectively, showing significantly different trends. In summary, the addition of polyphenol *co*-pigments to BHJ effectively increases the relative content of non-anthocyanin polyphenols in BHJ and enhances the bioactivity and nutritional value of the juice (Cao et al., 2023).

## 4 Conclusions

The findings demonstrated that polyphenol co-pigments greatly enhanced the color stability, anthocyanin retention, and antioxidant capacity of BHJ, and flavonoids were more effective than phenolic acids. The FTIR results pointed that anthocyanins possess unique flexible chemical structures that readily form complexes with co-pigments through non-covalent interactions such as π-π stacking, hydrogen bonding, and van der Waals forces, making them less susceptible to degradation. In addition, A total of 15 anthocyanins and 39 non-anthocyanin polyphenols were identified and quantified in BHJ, confirming that polyphenol co-pigments protect anthocyanins, specifically cyanidin-3-glucoside, from degradation to preserve color. Furthermore, polyphenol *co*-pigments significantly boosted the levels of non-anthocyanin polyphenols as well. Flavonoids such as quercetin, rutin, catechin, and EGCG exhibited stronger symchromization ability with anthocyanins compared to phenolic acids (gallic acid), providing better protection for the anthocyanins. This research proposes an effective approach to enhancing the color stability of BHJ while shedding light on the interaction mechanism between polyphenol co-pigments and anthocyanins. The results of the study can serve as a useful reference for leveraging polyphenol co-pigments as natural functional colorants in the beverage industry.

## CRediT authorship contribution statement

**Yifan Geng:** Writing – original draft, Conceptualization. **Kaojia Cui:** Writing – original draft. **Na Ding:** Data curation. **Houping Liu:** Formal analysis. **Junwei Huo:** Resources. **Xiaonan Sui:** Supervision. **Yan Zhang:** Writing – review & editing, Visualization, Conceptualization.

## Declaration of competing interest

The authors declare that they have no known competing financial interests or personal relationships that could have appeared to influence the work reported in this paper.

## Data Availability

Data will be made available on request.

## Appendix A Supplementary data

**Table S1.** HPLC-ESI-QTOF-MS^2^ of phenolic compounds of BHJ. Image 1
