# Supplementary material for: Polyphenol co-pigments enhanced the antioxidant capacity and color stability of blue honeysuckle juice during storage
Source: Food Chem X. 2024 Sep 20;24:101848. doi: 10.1016/j.fochx.2024.101848 (PMC11533655; doi:10.1016/j.fochx.2024.101848)

**Supplementary Material**

**Table S1.** HPLC-ESI-QTOF-MS^2^ of phenolic compounds of BHJ.

| Peak | Retention time (min) | Chemical formula | MW | Exact mass (m/z) | MS ([M]^+^/ [M-H]^-^) (m/z) | MS^2^ (m/z) | Error (ppm) | Tentative assignment | HPLC-DAD λmax (nm) |
| --- | --- | --- | --- | --- | --- | --- | --- | --- | --- |
| **1 Anthocyanins** | | | | | | | | | |
| A1 | 3.372 | C_30_H_27_O_13_^+^ | 595 | 595.1643 | 595.1656 | 287.0549/595.1649 | 0.21 | Cyanidin-3-(6'’-coumaroyl)-glucoside | 275, 518 |
| A2 | 3.867 | C_27_H_31_O_16_^+^ | 611 | 611.1592 | 611.1606 | 287.0556/449.1070/611.1600 | 1.35 | Cyanidin-3,5-diglucoside | 281, 519 |
| A3 | 5.095 | C_27_H_31_O_15_^+^ | 595 | 595.1633 | 595.1639 | 287.0560 | 0.72 | Cyanidin-3-rutinoside | 279, 514 |
| A4 | 7.438 | C_28_H_33_O_16_^+^ | 625 | 625.1750 | 625.1746 | 301.0707/463.1231/625.1756 | 0.59 | Peonidin-3,5-diglucoside | 279, 517 |
| A5 | 10.644 | C_21_H_21_O_11_^+^ | 449 | 449.1070 | 449.0444 | 287.0538 | 0.74 | Cyanidin-3-glucoside | 280, 517 |
| A6 | 14.723 | C_22_H_23_O_11_^+^ | 463 | 463.1226 | 463.1216 | 301.0708 | 0.30 | Peonidin-3-glucoside | 281, 506 |
| A7 | 16.479 | C_21_H_21_O_10_^+^ | 433 | 433.1125 | 433.1127 | 271.0600 | -0.34 | Pelargonidin-3-glucoside | 281, 508 |
| A8 | 17.156 | C_28_H_33_O_15_^+^ | 609 | 609.1798 | 609.1508 | 301.0713 | 0.90 | Peonidin-3-rutinoside | 280, 525 |
| A9 | 17.875 | C_20_H_19_O_10_^+^ | 419 | 419.0961 | 419.0944 | 287.0550 | -0.63 | Cyanidin-3-xyloside | 283, 525 |
| A10 | 20.961 | C_23_H_23_O_12_^+^ | 491 | 491.1168 | 491.1180 | 287.0545/491.1154 | 0.52 | Cyanidin-3-(6'’-acetyl)-glucoside | 276, 520 |
| A11 | 22.334 | C_26_H_29_O_14_^+^ | 597 | 597.1437 | 597.1438 | 303.0502 | -0.82 | Delphinidin-3-sambubioside | 274, 518 |
| A12 | 23.033 | C_27_H_31_O_16_^+^ | 611 | 611.1591 | 613.1463 | 305.0560/304.0539/467.1051 | 0.76 | Delphinidin-3-rutinoside | 265, 524 |
| A13 | 23.513 | C_26_H_29_O_15_^+^ | 581 | 581.1473 | 581.1468 | 287.0534/449.1239 | 0.18 | Cyanidin-3-sambubioside | 252, 518 |
| A14 | 23.892 | C_25_H_23_O_11_^+^ | 487 | 487.0835 | 487.0835 | 185.0420/325.0300/487.0848 | 0.64 | B-type vitisin peonidin-3-hexoside | 254, 526 |
| A15 | 25.394 | C_21_H_21_O_12_^+^ | 465 | 465.1017 | 465.1012 | 303.0501 | 0.58 | Delphinidin-3-glucoside | 252, 526 |
| **2 Flavonols and isoflavonols** | | | | | | | | | |
| 6 | 5.671 | C_16_H_12_O_7_ | 316 | 315.0733 | 315.0739 | 108.0207/152.0111 | 0.44 | Isorhamnetin | 226, 287 |
| 7 | 5.867 | C_27_H_30_O_16_ | 610 | 609.1496 | 609.1501 | 285.0412/447.0959 | -1.04 | Kaempferol-3-gentiobioside | 227, 280 |
| 11 | 7.437 | C_21_H_20_O_12_ | 464 | 463.0879 | 463.0893 | 283.0253/301.0350 | 2.59 | Quercetin-3-glucoside | 234 |
| 13 | 9.414 | C_27_H_30_O_15_ | 594 | 593.1543 | 593.1108 | 284.0344/285.0415 | -0.02 | Kaempferol-3-rutinoside | 235 |
| 19 | 14.450 | C_20_H_20_O_12_ | 434 | 433.1369 | 433.1372 | 225.0778/301.0361 | -0.07 | Quercetin-3-pentoside | 243 |
| 28 | 22.218 | C_33_H_40_O_21_ | 610 | 609.1511 | 609.1520 | 301.0365/609.1495 | 0.33 | Quercetin-3-rutinoside | 252, 356 |
| 30 | 23.080 | C_21_H_20_O_12_ | 464 | 463.0909 | 463.0913 | 255.0314/271.0257/301.0372 | 1.23 | Quercetin-3-galactoside | 261, 318 |
| 31 | 23.822 | C_20_H_20_O_12_ | 434 | 433.0785 | 433.0777 | 300.0284/301.0365 | 0.15 | Quercetin-3-xyloside | 252 |
| 33 | 24.924 | C_21_H_29_O_14_ | 506 | 505.0989 | 505.1009 | 300.0282/301.0369 | 4.75 | Quercetin-3-acetylhexoside | 257 |
| 35 | 26.570 | C_20_H_20_O_12_ | 434 | 433.1150 | 433.1147 | 271.0584/301.0371 | -0.14 | Quercetin-3-arabinoside | 243 |
| 37 | 29.599 | C_15_H_10_O_7_ | 302 | 301.0362 | 301.0362 | 151.0034/301.0360 | -0.53 | Quercetin | 244, 369 |
| **3 Flavanols** | | | | | | | | | |
| 15 | 11.148 | C_15_H_14_O_6_ | 290 | 289.0729 | 289.0723 | 109.0286/123.0450/289.0738 | -0.73 | (+)-Catechin | 236, 276 |
| 16 | 11.449 | C_15_H_14_O_6_ | 290 | 289.0720 | 289.0725 | 123.0434/203.0728/289.0740 | 2.89 | (-)-Catechin | 242, 285, 322 |
| 22 | 17.186 | C_22_H_18_O_11_ | 458 | 457.0783 | 457.1420 | 161.0246/169.0137/305.0668 | -0.38 | Epigallocatechin gallate | 219, 279 |
| 23 | 17.953 | C_15_H_14_O_6_ | 290 | 289.0360 | 289.0355 | 109.0282/151.0031/178.9990 | 1.04 | Epicatechin | 245, 274 |
| **4 Flavones** | | | | | | | | | |
| 8 | 6.246 | C_30_H_18_O_10_ | 538 | 537.1831 | 537.1852 | 213.0771/375.1301 | 3.41 | Amentoflavone | 232 |
| 36 | 29.389 | C_15_H_10_O_6_ | 286 | 285.0405 | 285.0410 | 133.0285/151.0036 | 2.53 | Luteolin | 244 |
| 39 | 32.497 | C_15_H_10_O_6_ | 286 | 285.0414 | 285.0416 | 285.0419 | 0.60 | Scutellarein | 250 |
| **5 Flavanones** | | | | | | | | | |
| 25 | 21.190 | C_27_H_32_O_15_ | 596 | 595.1338 | 595.1330 | 271.0262/300.0284/301.0363 | 0.51 | Neoeriocitrin | 262 |
| 38 | 32.027 | C_15_H_12_O_5_ | 272 | 271.0613 | 271.0624 | 119.0502/151.0008/271.0640 | 2.96 | Naringenin | 300 |
| **6 Phenolic acids** | | | | | | | | | |
| 1 | 2.513 | C_30_H_48_O_4_ | 472 | 471.1375 | 471.1367 | 317.0902/411.1176 | 0.23 | Corosolic acid | 207, 211, 261 |
| 2 | 2.718 | C_7_H_12_O_6_ | 192 | 191.0567 | 191.0564 | 85.0288/191.0563 | -2.07 | Quinic acid | 208, 225 |
| 3 | 3.234 | C_6_H_8_O_7_ | 192 | 191.0203 | 191.0007 | 87.0077/111.0079 | 0.50 | Citric acid | 209, 224 |
| 4 | 3.859 | C_16_H_24_O_10_ | 376 | 375.1308 | 375.1314 | 213.0770 | 0.68 | 8-Epiloganic acid | 196, 224 |
| 5 | 4.467 | C_7_H_6_O_5_ | 170 | 169.0874 | 169.0872 | 95.0475/123.0826 | -1.82 | Gallic acid | 284 |
| 10 | 6.910 | C_16_H_18_O_9_ | 354 | 353.0889 | 353.0890 | 135.0453/179.0360/191.0572 | 0.65 | Neochlorogenic acid | 229, 317 |
| 12 | 7.672 | C_7_H_6_O_4_ | 154 | 153.0197 | 153.0197 | 109.0288/108.0211 | 0.00 | Protocatechuic acid | 238 |
| 14 | 9.967 | C_7_H_6_O_4_ | 316 | 315.0725 | 315.0744 | 153.0194/151.0034/125.0227 | 2.37 | Protocatechuic acid hexoside | 239 |
| 17 | 11.483 | C_7_H_6_O_3_ | 138 | 137.0244 | 137.0241 | 108.0205/137.0237 | -1.34 | Protocatechuic aldehyde | 243, 276 |
| 20 | 15.612 | C_9_H_8_O_4_ | 180 | 179.0350 | 179.0346 | 89.0350/135.0442/134.0361 | 1.44 | Caffeic acid | 252 |
| 21 | 16.813 | C_16_H_18_O_9_ | 354 | 353.0877 | 353.0886 | 191.0551 | 4.86 | Chlorogenic acid | 239, 321 |
| 24 | 19.580 | C_17_H_20_O_9_ | 368 | 367.1045 | 367.1039 | 191.0550 | 0.46 | Feruloylquinic acid | 249, 317 |
| 27 | 21.740 | C_18_H_16_O_8_ | 360 | 359.1358 | 359.1367 | 153.0917/197.0819 | 0.05 | Rosmarinic acid | 261, 347 |
| 29 | 22.376 | C_22_H_34_O_3_ | 346 | 345.1568 | 345.1571 | 59.0122/165.0917 | 2.06 | Ginkgolic Acid (C15:1) | 260 |
| 32 | 24.797 | C_25_H_24_O_12_ | 516 | 515.1210 | 515.1210 | 179.0351/191.0536 | 0.19 | 3,5-di-Caffeoylquinic acid | 286 |
| **7 Procyanidin dimer** | | | | | | | | | |
| 18 | 14.000 | C_30_H_26_O_12_ | 578 | 577.1387 | 577.1390 | 125.0235/289.0731/407.0791 | 0.44 | Procyanidin B1 | 241 |
| **8 Other polyphenolic compounds** | | | | | | | | | |
| 9 | 6.642 | C_21_H_20_O_6_ | 368 | 367.0681 | 367.0680 | 176.0115/205.0148 | 0.48 | Curcumin | 231, 278 |
| 26 | 21.335 | C_28_H_36_O_15_ | 612 | 611.1567 | 611.1580 | 271.0258/301.0335 | 0.71 | Neohesperidin dihydrochalcone | 252, 356 |
| 34 | 25.785 | C_21_H_24_O_10_ | 436 | 435.1295 | 435.1306 | 167.0347/273.0774 | 3.07 | Phloridzin | 249, 314, 337 |

**Table S2.** Color parameters, TAC, and antioxidant capacity of BHJ.

| Storage time | Material | Ratio | L* | a* | b* | TAC/  (C3G mg/g DW) | DPPH/  (μmol TE/g DW) | ABTS/  (μmol TE/g DW) | FRAP/  (μmol Fe^2+^/g DW) |
| --- | --- | --- | --- | --- | --- | --- | --- | --- | --- |
| 0d | ACNs: GA | 1:0 | 23.08±0.02^Ca^ | 1.83±0.02^Bc^ | -0.15±0.01^Dd^ | 18.65±0.17^Ab^ | 45.21±0.47^Ae^ | 8.59±0.29^Ad^ | 353.19±4.23^Ae^ |
|  |  | 1:1 | 23.10±0.01^Aa^ | 1.98±0.01^Aa^ | -0.12±0.01^Dc^ | 18.78±0.16^Ab^ | 50.27±0.82^Ad^ | 8.92±0.24^Ad^ | 384.18±11.00^Ad^ |
|  |  | 1:5 | 22.91±0.02^Ec^ | 1.88±0.01^Bb^ | 0.03±0.03^Aa^ | 19.53±0.35^Aa^ | 58.69±1.40^Ac^ | 10.07±0.21^Ac^ | 480.90±12.81^Ac^ |
|  |  | 1:10 | 23.08±0.03^Aa^ | 2.00±0.01^Aa^ | -0.38±0.02^Ee^ | 19.66±0.78^Aa^ | 69.90±0.78^Bb^ | 10.78±0.19^Ab^ | 605.14±19.90^Ab^ |
|  |  | 1:20 | 23.03±0.02^Cb^ | 1.90±0.04^Bb^ | -0.04±0.02^Eb^ | 19.73±0.12^Aa^ | 93.34±0.53^Aa^ | 12.19±0.22^Aa^ | 789.61±12.70^Aa^ |
|  | ACNs: QTI | 1:0 | 23.01±0.03^BCd^ | 1.82±0.01^Be^ | -0.15±0.01^Db^ | 22.54±0.41^Aa^ | 50.21±0.86^Ae^ | 7.86±0.23^Ad^ | 373.89±13.64^Ad^ |
|  |  | 1:1 | 23.06±0.01^Cd^ | 2.03±0.02^Ac^ | -0.02±0.01^Ca^ | 22.68±0.57^Aa^ | 52.14±0.74^Ad^ | 8.45±0.26^Ac^ | 390.55±11.68^Acd^ |
|  |  | 1:5 | 23.12±0.02^Fc^ | 1.88±0.02^Ed^ | -0.34±0.01^Fc^ | 22.74±0.35^Aa^ | 55.03±0.74^Ac^ | 9.24±0.04^Ab^ | 415.45±26.59^Abc^ |
|  |  | 1:10 | 23.24±0.02^Eb^ | 4.06±0.01^Ab^ | -0.36±0.03^Fc^ | 23.38±1.00^Aa^ | 60.01±0.30^Ab^ | 9.71±0.23^Aa^ | 442.67±18.71^Ab^ |
|  |  | 1:20 | 25.22±0.09^Fa^ | 6.45±0.05^Aa^ | -0.35±0.03^Gc^ | 23.59±0.94^Aa^ | 68.34±1.40^Ca^ | 10.08±0.35^Aa^ | 484.37±22.23^Aa^ |
|  | ACNs: RUT | 1:0 | 25.91±0.01^Ed^ | 1.84±0.01^Aa^ | 0.61±0.02^Da^ | 23.40±0.63^Aa^ | 53.11±0.19^Ac^ | 7.30±0.14^Dc^ | 377.03±2.92^Abc^ |
|  |  | 1:1 | 26.03±0.00^Ac^ | 1.60±0.01^Ac^ | 0.48±0.01^Fb^ | 23.91±0.40^Aa^ | 52.90±0.56^Ac^ | 9.32±0.07^Ca^ | 422.21±17.54^Ba^ |
|  |  | 1:5 | 26.12±0.00^Aa^ | 1.65±0.01^Ab^ | 0.41±0.01^Fd^ | 24.28±0.66^Aa^ | 60.43±0.67^Ab^ | 9.04±0.03^Ca^ | 383.60±4.63^ABb^ |
|  |  | 1:10 | 26.10±0.01^Ab^ | 1.58±0.01^Ad^ | 0.45±0.01^Gc^ | 23.88±0.92^Aa^ | 60.32±0.56^Ab^ | 8.60±0.21^Bb^ | 360.43±10.10^Cc^ |
|  |  | 1:20 | 26.03±0.01^Cc^ | 1.52±0.01^Ae^ | 0.46±0.02^Fc^ | 24.16±0.40^Aa^ | 62.79±0.19^Aa^ | 8.69±0.29^Cb^ | 411.01±3.54^Ba^ |
|  | ACNs: CAT | 1:0 | 25.91±0.01^Ee^ | 1.84±0.01^Ad^ | 0.61±0.02^Dc^ | 23.40±0.63^Ab^ | 53.11±0.19^Ac^ | 7.30±0.14^Dd^ | 377.03±2.92^Ab^ |
|  |  | 1:1 | 26.27±0.01^Aa^ | 2.70±0.01^Aa^ | 0.76±0.01^Ea^ | 17.73±0.23^Ae^ | 46.66±0.49^Bd^ | 7.51±0.05^BCd^ | 232.23±9.85^Cd^ |
|  |  | 1:5 | 26.16±0.03^Ab^ | 2.36±0.03^Ab^ | 0.63±0.01^Eb^ | 18.83±0.20^Ad^ | 53.22±0.00^Ac^ | 7.97±0.07^Dc^ | 269.69±6.59^Cc^ |
|  |  | 1:10 | 25.98±0.01^Ad^ | 2.00±0.01^Ac^ | 0.63±0.01^Gb^ | 20.57±0.13^Ac^ | 62.25±0.32^Bb^ | 8.57±0.16^Db^ | 389.00±2.92^Cb^ |
|  |  | 1:20 | 26.14±0.01^Ac^ | 1.80±0.01^Ae^ | 0.46±0.01^Fd^ | 24.57±0.44^Aa^ | 76.02±1.04^Aa^ | 9.53±0.28^Da^ | 462.75±13.82^Ba^ |
|  | ACNs: EGCG | 1:0 | 25.91±0.01^Ec^ | 1.84±0.01^Ab^ | 0.61±0.02^Db^ | 23.40±0.63^Ac^ | 53.11±0.19^Ae^ | 7.30±0.14^De^ | 377.03±2.92^Ad^ |
|  |  | 1:1 | 25.92±0.02^Bc^ | 1.66±0.02^Ac^ | 0.57±0.01^Fc^ | 25.60±0.47^Ab^ | 58.49±0.37^Ad^ | 8.05±0.03^CDd^ | 385.53±10.76^Ad^ |
|  |  | 1:5 | 26.03±0.02^Ab^ | 1.42±0.01^Be^ | 0.40±0.03^Dd^ | 26.65±0.51^Aa^ | 62.68±0.37^Bc^ | 8.57±0.05^Dc^ | 441.51±3.48^Bc^ |
|  |  | 1:10 | 25.81±0.01^Bd^ | 1.48±0.02^Ad^ | 0.62±0.02^Eab^ | 26.32±0.99^Aa^ | 65.91±0.49^Cb^ | 9.16±0.18^CDb^ | 464.30±8.54^Cb^ |
|  |  | 1:20 | 26.07±0.01^Ba^ | 1.91±0.01^Aa^ | 0.64±0.01^Ea^ | 21.89±0.66^Ad^ | 78.39±0.56^Ba^ | 9.97±0.20^Ba^ | 506.38±8.11^Ca^ |
| 4d | ACNs: GA | 1:0 | 22.99±0.01^Ec^ | 1.89±0.02^Ac^ | 0.07±0.00^Ab^ | 15.67±0.26^Bc^ | 42.18±1.26^Be^ | 8.10±0.26^Bd^ | 342.18±6.97^Ae^ |
|  |  | 1:1 | 23.06±0.01^Ba^ | 1.89±0.02^Bc^ | 0.03±0.01^BCc^ | 16.20±0.23^Bbc^ | 46.65±0.31^Cd^ | 8.32±0.16^Bd^ | 365.06±3.34^Bd^ |
|  |  | 1:5 | 23.03±0.03^CDb^ | 1.93±0.01^Ab^ | -0.07±0.02^Cd^ | 16.59±0.59^Bab^ | 55.74±0.44^Bc^ | 9.22±0.08^Bc^ | 435.43±18.35^BCc^ |
|  |  | 1:10 | 22.95±0.01^Ed^ | 1.84±0.01^Bd^ | 0.06±0.01^Ab^ | 16.64±0.23^Bab^ | 67.30±1.27^CDb^ | 10.03±0.38^Bb^ | 558.80±6.97^Bb^ |
|  |  | 1:20 | 23.02±0.01^Cb^ | 2.04±0.05^Aa^ | 0.10±0.01^Aa^ | 16.91±0.12^Ba^ | 88.11±2.40^Ba^ | 11.06±0.23^BCa^ | 749.93±12.38^Ba^ |
|  | ACNs: QTI | 1:0 | 22.99±0.01^Ce^ | 1.89±0.02^Ad^ | 0.07±0.00^Bb^ | 17.21±0.24^Bc^ | 45.72±1.43^Be^ | 6.54±0.34^Bd^ | 357.23±9.42^Bd^ |
|  |  | 1:1 | 23.06±0.01^Cd^ | 1.97±0.01^Bc^ | 0.05±0.03^Ab^ | 17.73±0.18^Bc^ | 48.42±2.31^Bd^ | 7.36±0.61^Cc^ | 372.01±11.41^Bd^ |
|  |  | 1:5 | 23.11±0.02^Fc^ | 1.84±0.02^Ed^ | -0.09±0.01^Bc^ | 18.99±0.13^Bb^ | 52.43±1.43^Bc^ | 8.10±0.58^BCb^ | 389.68±8.15^Bc^ |
|  |  | 1:10 | 24.57±0.07^Cb^ | 3.84±0.13^Bb^ | -0.16±0.02^Ed^ | 19.72±0.68^Ba^ | 56.21±1.27^BCb^ | 8.79±0.24^Ba^ | 414.00±2.74^Bb^ |
|  |  | 1:20 | 26.12±0.02^Ea^ | 6.24±0.02^Ba^ | 0.55±0.02^Fa^ | 20.13±0.10^Ba^ | 64.84±1.19^Ca^ | 9.13±0.48^Ba^ | 431.67±15.51^Ba^ |
|  | ACNs: RUT | 1:0 | 26.02±0.01^Bb^ | 1.75±0.02^Ba^ | 0.58±0.02^Ec^ | 17.26±0.17^Bc^ | 46.59±0.48^De^ | 7.92±0.08^Cc^ | 318.63±10.51^Cd^ |
|  |  | 1:1 | 25.91±0.01^Cd^ | 1.56±0.02^Bb^ | 0.64±0.01^Ea^ | 19.66±0.56^Ba^ | 51.42±0.18^Ad^ | 9.52±0.10^Ca^ | 475.01±15.43^Aa^ |
|  |  | 1:5 | 25.90±0.04^Bd^ | 1.52±0.01^Bc^ | 0.61±0.01^Eb^ | 18.24±0.20^Bb^ | 53.74±0.00^Cc^ | 8.73±0.32^Db^ | 392.77±8.22^Ac^ |
|  |  | 1:10 | 25.95±0.01^Bc^ | 1.50±0.02^Bd^ | 0.57±0.02^Fc^ | 19.17±0.22^Ba^ | 55.63±0.00^Cb^ | 8.67±0.09^Bb^ | 421.73±6.59^Ab^ |
|  |  | 1:20 | 26.08±0.01^Aa^ | 1.42±0.02^Be^ | 0.52±0.02^Dd^ | 17.79±0.32^Bbc^ | 58.46±0.83^BCa^ | 8.50±0.18^Cb^ | 438.33±3.34^Ab^ |
|  | ACNs: CAT | 1:0 | 26.02±0.01^Bb^ | 1.75±0.02^Bd^ | 0.58±0.02^Ee^ | 17.26±0.17^Bb^ | 46.59±0.48^Dd^ | 7.92±0.08^Cc^ | 318.63±10.51^Cd^ |
|  |  | 1:1 | 26.12±0.01^Ba^ | 2.57±0.02^Ba^ | 0.96±0.02^Da^ | 13.60±0.49^Bd^ | 41.55±0.48^Ce^ | 7.24±0.21^CDd^ | 308.21±4.68^Ad^ |
|  |  | 1:5 | 26.11±0.01^Ba^ | 2.30±0.01^Bb^ | 0.82±0.01^Db^ | 15.32±0.59^Bc^ | 49.85±0.18^Bc^ | 8.93±0.39^Bb^ | 358.79±3.48^Ac^ |
|  |  | 1:10 | 25.90±0.01^Cd^ | 1.87±0.02^Bc^ | 0.78±0.01^Ec^ | 17.33±0.09^Bb^ | 57.52±0.00^Db^ | 9.46±0.43^Ca^ | 414.78±4.82^Ab^ |
|  |  | 1:20 | 25.98±0.01^Cc^ | 1.76±0.01^Bd^ | 0.72±0.01^Dd^ | 18.35±0.11^Ba^ | 69.29±0.48^Ca^ | 9.91±0.18^Ca^ | 497.41±6.79^Aa^ |
|  | ACNs: EGCG | 1:0 | 26.02±0.01^Bb^ | 1.75±0.02^Bb^ | 0.58±0.02^Ec^ | 17.26±0.17^Bc^ | 46.59±0.48^De^ | 7.92±0.08^Cd^ | 318.63±10.51^Ce^ |
|  |  | 1:1 | 25.81±0.04^Cc^ | 1.55±0.02^Bc^ | 0.67±0.02^Da^ | 20.16±0.54^Ba^ | 52.37±0.48^Dd^ | 7.86±0.31^Dd^ | 393.92±2.68^Ad^ |
|  |  | 1:5 | 26.02±0.02^Ab^ | 1.45±0.02^Ad^ | 0.60±0.02^Cbc^ | 21.01±0.22^Ba^ | 61.72±0.36^BCc^ | 8.31±0.10^Dc^ | 466.13±4.07^Ac^ |
|  |  | 1:10 | 25.78±0.03^Cc^ | 1.28±0.03^BCe^ | 0.68±0.02^Da^ | 18.46±0.70^Bb^ | 67.92±0.32^Bb^ | 9.02±0.19^DEb^ | 541.81±12.53^Ab^ |
|  |  | 1:20 | 26.26±0.06^Aa^ | 1.80±0.01^Ba^ | 0.62±0.01^Fb^ | 15.20±0.87^Bd^ | 79.48±0.66^Ba^ | 9.95±0.16^Ba^ | 574.25±7.60^Aa^ |
| 8d | ACNs: GA | 1:0 | 23.05±0.02^Dc^ | 1.87±0.03^Ab^ | 0.09±0.02^Aa^ | 13.59±0.26^Cc^ | 40.71±1.66^Be^ | 7.94±0.19^Be^ | 306.45±6.76^Ce^ |
|  |  | 1:1 | 23.01±0.01^Cd^ | 1.76±0.02^Cd^ | 0.06±0.02^Ab^ | 14.04±0.36^Cbc^ | 44.30±0.25^Dd^ | 8.29±0.26^Bd^ | 334.89±3.46^Cd^ |
|  |  | 1:5 | 23.20±0.01^Aa^ | 1.95±0.01^Aa^ | -0.05±0.02^BCd^ | 14.12±0.05^Cbc^ | 51.72±0.39^Dc^ | 8.93±0.05^Cc^ | 429.17±11.23^Cc^ |
|  |  | 1:10 | 23.02±0.01^BCd^ | 1.79±0.03^Cc^ | 0.07±0,01^Ab^ | 14.45±0.37^Cab^ | 66.31±1.67^Db^ | 10.32±0.35^ABb^ | 534.78±5.56^BCb^ |
|  |  | 1:20 | 23.15±0.01^Ab^ | 1.79±0.02^Cc^ | -0.01±0.02^Dc^ | 14.66±0.21^Ca^ | 84.50±1.60^Ca^ | 10.70±0.11^Ca^ | 722.56±7.89^Ca^ |
|  | ACNs: QTI | 1:0 | 23.05±0.02^Ae^ | 1.87±0.03^Ad^ | 0.09±0.02^ABb^ | 15.34±0.46^Cd^ | 41.28±1.31^eC^ | 6.52±0.27^Bd^ | 323.45±7.12^Cd^ |
|  |  | 1:1 | 23.09±0.02^Bd^ | 1.87±0.01^Cd^ | 0.03±0.01^ABc^ | 16.01±0.27^Cc^ | 45.78±1.00^Bd^ | 7.08±0.18^CDc^ | 341.56±8.12^Cd^ |
|  |  | 1:5 | 23.26±0.02^Ec^ | 1.97±0.02^Dc^ | 0.10±0.02^Ab^ | 16.42±0.36^Cb^ | 49.61±0.81^Dc^ | 7.83±0.23^Cb^ | 370.69±8.93^Cc^ |
|  |  | 1:10 | 24.47±0.02^Db^ | 3.73±0.01^Cb^ | -0.07±0.01^Dd^ | 16.59±0.18^Cb^ | 54.84±0.90^Cb^ | 8.11±0.22^Cb^ | 404.13±7.35^Bb^ |
|  |  | 1:20 | 26.48±0.02^Da^ | 6.26±0.02^Ba^ | 0.73±0.02^Ea^ | 17.07±0.23^Ca^ | 63.89±0.78^Ca^ | 8.83±0.53^Ba^ | 420.66±8.95^Ba^ |
|  | ACNs: RUT | 1:0 | 26.06±0.00^Aa^ | 1.59±0.01^Ca^ | 0.55±0.01^Fd^ | 14.71±0.59^Cc^ | 43.18±0.16^Fe^ | 7.34±0.17^Db^ | 355.89±1.77^Be^ |
|  |  | 1:1 | 25.93±0.01^Bb^ | 1.48±0.01^Cb^ | 0.68±0.01^Da^ | 16.91±0.13^Ca^ | 44.75±0.28^Cd^ | 8.55±0.08^Da^ | 369.79±1.77^Cd^ |
|  |  | 1:5 | 25.90±0.02^Bc^ | 1.49±0.02^Cb^ | 0.65±0.01^Db^ | 15.75±0.70^Cb^ | 48.78±0.16^Dc^ | 8.43±0.17^Ea^ | 378.29±0.67^Bc^ |
|  |  | 1:10 | 25.87±0.01^Dd^ | 1.46±0.01^Cc^ | 0.64±0.01^Dc^ | 15.34±0.40^Cbc^ | 49.43±0.28^Eb^ | 8.64±0.15^Ba^ | 390.64±0.67^Bb^ |
|  |  | 1:20 | 26.05±0.02^Ba^ | 1.35±0.01^Cd^ | 0.50±0.01^Ee^ | 15.32±0.53^Cbc^ | 53.47±0.32^Da^ | 8.71±0.16^Ca^ | 441.23±6.45^Aa^ |
|  | ACNs: CAT | 1:0 | 26.06±0.00^Aa^ | 1.59±0.01^Ce^ | 0.55±0.01^Fe^ | 14.71±0.59^Ca^ | 43.18±0.16^Fd^ | 7.34±0.17^Dc^ | 355.89±1.77^Bc^ |
|  |  | 1:1 | 25.99±0.02^Db^ | 2.59±0.01^Ba^ | 1.12±0.03^Ba^ | 10.85±0.59^Cc^ | 39.05±0.16^De^ | 7.08±0.21^Dc^ | 257.04±15.06^Be^ |
|  |  | 1:5 | 25.92±0.01^Dc^ | 2.28±0.04^Bb^ | 1.01±0.01^Bb^ | 13.16±0.32^Cb^ | 46.67±0.28^Cc^ | 8.37±0.10^Cb^ | 340.45±1.16^Bd^ |
|  |  | 1:10 | 25.92±0.01^Bc^ | 1.86±0.01^Bc^ | 0.76±0.02^Fc^ | 14.65±0.45^Ca^ | 54.94±0.00^Eb^ | 9.60±0.15^Ca^ | 402.23±3.54^Bb^ |
|  |  | 1:20 | 26.06±0.01^Ba^ | 1.70±0.02^Cd^ | 0.67±0.02^Ed^ | 15.00±0.36^Ca^ | 63.56±0.64^Da^ | 9.82±0.15^CDa^ | 500.69±1.34^Aa^ |
|  | ACNs: EGCG | 1:0 | 26.06±0.00^Aa^ | 1.59±0.01^Cb^ | 0.55±0.01^Fd^ | 14.71±0.59^Cbc^ | 43.18±0.16^Fe^ | 7.34±0.17^Dd^ | 355.89±1.77^Bc^ |
|  |  | 1:1 | 26.07±0.01^Aa^ | 1.43±0.01^Cc^ | 0.62±0.01^Eb^ | 15.91±0.26^Cb^ | 47.50±0.28^Ed^ | 8.35±0.18^BCc^ | 355.12±10.76^Bc^ |
|  |  | 1:5 | 25.85±0.01^Bc^ | 1.27±0.01^Ce^ | 0.59±0.02^Cc^ | 17.92±0.71^Ca^ | 54.11±0.55^Ec^ | 9.20±0.10^Cb^ | 436.21±13.82^Bb^ |
|  |  | 1:10 | 25.87±0.01^Ab^ | 1.30±0.02^Bd^ | 0.59±0.01^Fc^ | 16.93±0.83^Bab^ | 60.99±0.73^Db^ | 9.54±0.20^BCab^ | 483.70±5.22^Bb^ |
|  |  | 1:20 | 26.07±0.03^Ba^ | 1.78±0.01^Ba^ | 0.74±0.01^Da^ | 13.76±0.19^Cc^ | 66.69±0.57^Ea^ | 9.82±0.27^Ba^ | 546.64±6.45^Ba^ |
| 12d | ACNs: GA | 1:0 | 22.98±0.01^Ec^ | 1.69±0.02^Ca^ | 0.04±0.02^Ba^ | 10.61±0.26^Dc^ | 45.31±0.44^Ae^ | 8.31±0.10^ABc^ | 288.90±16.88^Dd^ |
|  |  | 1:1 | 23.00±0.01^Cb^ | 1.60±0.01^Dc^ | 0.04±0.02^ABa^ | 11.13±0.42^Dbc^ | 48.13±1.23^Bd^ | 7.55±0.07^Dd^ | 308.88±7.39^Dd^ |
|  |  | 1:5 | 23.05±0.01^Ca^ | 1.64±0.01^Cb^ | 0.04±0.02^Aa^ | 11.38±0.17^Dabc^ | 57.75±0.86^Ac^ | 8.43±0.09^Dc^ | 417.48±3.46^CDc^ |
|  |  | 1:10 | 23.04±0.03^Ba^ | 1.64±0.02^Db^ | -0.04±0.02^Cb^ | 11.47±0.39^Dab^ | 72.15±0.78^Ab^ | 9.04±0.23^Cb^ | 519.99±11.48^CDb^ |
|  |  | 1:20 | 23.06±0.01^Ba^ | 1.69±0.02^Da^ | -0.03±0.01^Eb^ | 11.96±0.64^Da^ | 90.69±0.92^ABa^ | 11.30±0.03^Ba^ | 691.43±36.18^CDa^ |
|  | ACNs: QTI | 1:0 | 23.05±0.03^Ae^ | 1.71±0.02^Ce^ | 0.10±0.02^Ab^ | 12.67±0.48^Dc^ | 44.13±0.88^Be^ | 7.41±0.34^Ac^ | 272.89±7.23^Ee^ |
|  |  | 1:1 | 23.12±0.01^Ad^ | 1.80±0.01^Dd^ | 0.01±0.01^Bc^ | 13.03±0.50^Dbc^ | 47.90±1.48^Bd^ | 7.92±0.40^Bb^ | 295.85±7.76^Ed^ |
|  |  | 1:5 | 23.57±0.02^Dc^ | 2.55±0.04^Bc^ | -0.07±0.01^Bd^ | 13.68±0.59^Dab^ | 50.87±0.93^Cc^ | 8.50±0.08^Ba^ | 356.66±9.75^Cc^ |
|  |  | 1:10 | 24.58±0.02^Cb^ | 3.74±0.02^Cb^ | 0.03±0.02^Cc^ | 14.02±0.35^Da^ | 59.40±1.82^Ab^ | 5.94±0.09^Ed^ | 375.48±7.60^Cb^ |
|  |  | 1:20 | 26.48±0.02^Da^ | 6.25±0.09^Ba^ | 0.85±0.03^Da^ | 14.35±0.13^Da^ | 71.76±0.31^Ba^ | 7.98±0.18^Cb^ | 389.97±7.26^Ca^ |
|  | ACNs: RUT | 1:0 | 25.96±0.01^Cab^ | 1.55±0.01^Da^ | 0.58±0.01^Ec^ | 11.19±0.46^Dc^ | 44.26±0.76^Ed^ | 7.50±0.31^Dab^ | 305.12±3.48^Dbc^ |
|  |  | 1:1 | 25.94±0.00^Bb^ | 1.43±0.01^Db^ | 0.62±0.01^Eb^ | 13.09±0.73^Da^ | 44.84±0.00^Ccd^ | 7.65±0.15^Fab^ | 297.39±3.34^Ec^ |
|  |  | 1:5 | 25.81±0.03^Cd^ | 1.43±0.02^Db^ | 0.71±0.01^Ca^ | 12.74±0.24^Dab^ | 46.86±0.29^Ebc^ | 7.95±0.14^Fa^ | 338.32±9.85^Ca^ |
|  |  | 1:10 | 25.91±0.00^Cc^ | 1.39±0.01^Dc^ | 0.61±0.01^Eb^ | 12.72±0.27^Dab^ | 47.72±0.00^Fb^ | 7.66±0.15^Cab^ | 308.98±0.67^Db^ |
|  |  | 1:20 | 25.97±0.01^Da^ | 1.31±0.01^Dd^ | 0.54±0.01^Dd^ | 12.18±0.46^Db^ | 59.76±2.92^ABa^ | 7.47±0.31^Db^ | 341.41±4.07^Ca^ |
|  | ACNs: CAT | 1:0 | 25.96±0.01^Cc^ | 1.55±0.01^De^ | 0.58±0.01^Ee^ | 11.19±0.46^Dab^ | 44.26±0.76^Ec^ | 7.50±0.31^Db^ | 305.12±3.48^Dc^ |
|  |  | 1:1 | 26.03±0.02^Ca^ | 2.49±0.02^Ca^ | 1.06±0.01^Ca^ | 9.16±0.17^Dc^ | 35.11±0.33^Ee^ | 5.98±0.21^Ec^ | 233.68±3.54^Ce^ |
|  |  | 1:5 | 26.00±0.01^Cb^ | 2.17±0.01^Cb^ | 0.98±0.02^Cb^ | 10.75±0.30^Db^ | 42.62±0.33^Dd^ | 7.39±0.08^Eb^ | 254.15±4.01^Dd^ |
|  |  | 1:10 | 25.76±0.01^Fd^ | 1.79±0.01^Cc^ | 0.87±0.01^Bc^ | 12.09±0.30^Da^ | 50.32±0.76^Fb^ | 8.09±0.23^Ea^ | 339.87±6.45^Db^ |
|  |  | 1:20 | 25.99±0.03^Cb^ | 1.60±0.02^Dd^ | 0.71±0.01^Dd^ | 11.91±0.25^Da^ | 63.31±0.76^Da^ | 7.88±0.07^Ea^ | 407.83±14.67^Ca^ |
|  | ACNs: EGCG | 1:0 | 25.96±0.01^Ca^ | 1.55±0.01^Db^ | 0.58±0.01^Ed^ | 11.19±0.46^Dbc^ | 44.26±0.76^Ee^ | 7.50±0.31^Db^ | 305.12±3.48^Dd^ |
|  |  | 1:1 | 25.75±0.01^Db^ | 1.39±0.02^Dc^ | 0.73±0.02^Cb^ | 12.59±0.39^Db^ | 47.43±0.29^Ed^ | 7.16±0.05^Ec^ | 311.68±12.76^Cd^ |
|  |  | 1:5 | 25.69±0.02^Dc^ | 1.25±0.01^Dd^ | 0.73±0.02^ABb^ | 13.47±0.49^Da^ | 54.94±0.29^Ec^ | 7.54±0.22^Eb^ | 380.02±5.22^Cc^ |
|  |  | 1:10 | 25.71±0.03^Dc^ | 1.24±0.01^Dd^ | 0.69±0.01^Dc^ | 13.08±0.51^Cab^ | 59.28±0.76^Db^ | 8.69±0.12^Ea^ | 397.01±2.68^Db^ |
|  |  | 1:20 | 25.97±0.01^Ca^ | 1.73±0.01^Ca^ | 0.78±0.02^Ca^ | 10.46±0.22^Dc^ | 69.77±0.33^Da^ | 8.88±0.03^Ca^ | 465.74±0.67^Da^ |
| 16d | ACNs: GA | 1:0 | 23.08±0.02^Ca^ | 1.69±0.01^Ca^ | -0.01±0.02^Cc^ | 9.18±0.26^Ec^ | 40.90±0.71^Be^ | 7.54±0.04^Ce^ | 329.59±1.11^Bd^ |
|  |  | 1:1 | 22.95±0.01^Dd^ | 1.56±0.02^Ec^ | 0.02±0.02^BCb^ | 9.28±0.47^Ec^ | 44.42±0.40^Dd^ | 8.27±0.08^Bd^ | 338.27±15.20^Cd^ |
|  |  | 1:5 | 23.00±0.02^Db^ | 1.61±0.01^Db^ | -0.16±0.01^Ee^ | 9.60±0.28^Ebc^ | 55.84±0.41^Bc^ | 8.80±0.27^Cc^ | 409.22±17.18^Dc^ |
|  |  | 1:10 | 22.98±0.01^Dc^ | 1.63±0.02^Db^ | -0.10±0.02^Dd^ | 9.89±0.23^Eab^ | 67.19±0.45^CDb^ | 10.52±0.24^ABb^ | 502.76±22.29^Db^ |
|  |  | 1:20 | 22.83±0.01^Fe^ | 1.61±0.03^Eb^ | 0.04±0.01^Ca^ | 10.24±0.24^Ea^ | 89.88±0.26^Ba^ | 11.40±0.26^Ba^ | 684.34±12.13^Da^ |
|  | ACNs: QTI | 1:0 | 22.89±0.02^De^ | 1.59±0.01^De^ | -0.01±0.02^Cc^ | 10.77±0.24^Ec^ | 43.96±0.76^Be^ | 6.77±0.13^Bd^ | 301.77±4,34^Db^ |
|  |  | 1:1 | 23.00±0.02^Dd^ | 1.69±0.01^Ed^ | -0.03±0.02^Cc^ | 11.08±0.75^Ebc^ | 47.37±0.45^Bd^ | 7.27±0.09^Cc^ | 315.69±2.19^Db^ |
|  |  | 1:5 | 23.71±0.03^Cc^ | 2.54±0.03^Bc^ | -0.26±0.01^Ed^ | 11.59±0.41^Eab^ | 49.29±0.52^Dc^ | 7.59±0.11^Cc^ | 310.76±8.51^Db^ |
|  |  | 1:10 | 24.60±0.03^Cb^ | 3.55±0.04^Db^ | 0.11±0.01^Bb^ | 11.69±0.40^Eab^ | 58.08±0.32^ABb^ | 8.03±0.24^CDb^ | 340.01±13.63^Da^ |
|  |  | 1:20 | 26.89±0.03^Ca^ | 5.86±0.06^Ca^ | 0.89±0.03^Ca^ | 11.91±0.07^Ea^ | 67.83±1.14^Ba^ | 9.07±0.42^Ba^ | 354.20±15.90^Da^ |
|  | ACNs: RUT | 1:0 | 25.93±0.03^Da^ | 1.43±0.02^Ea^ | 0.64±0.02^Cb^ | 9.54±0.18^Ec^ | 46.63±0.21^De^ | 7.95±0.14^Cc^ | 228.47±4.39^Ge^ |
|  |  | 1:1 | 25.74±0.02^Dd^ | 1.37±0.03^Ec^ | 0.75±0.03^Ca^ | 11.23±0.17^Ea^ | 48.48±0.53^Bd^ | 8.14±0.11^Eb^ | 241.98±2.92^Gd^ |
|  |  | 1:5 | 25.93±0.03^Ba^ | 1.30±0.03^Ed^ | 0.65±0.03^Db^ | 10.93±0.34^Ea^ | 49.64±0.00^Dc^ | 8.72±0.05^Da^ | 254.34±3.48^Ec^ |
|  |  | 1:10 | 25.82±0.01^Ec^ | 1.40±0.02^Db^ | 0.74±0.02^Ca^ | 10.43±0.35^Eb^ | 51.48±0.20^Db^ | 8.60±0.08^Ba^ | 265.54±4.82^Fb^ |
|  |  | 1:20 | 25.86±0.03^Eb^ | 1.27±0.02^Ed^ | 0.65±0.02^Cb^ | 10.12±0.06^Eb^ | 56.22±0.00^CDa^ | 8.57±0.10^Ca^ | 297.20±3.48^Ea^ |
|  | ACNs: CAT | 1:0 | 25.93±0.03^Db^ | 1.43±0.02^Ee^ | 0.64±0.02^Ce^ | 9.54±0.18^Eb^ | 46.63±0.21^Dd^ | 7.95±0.14^Cd^ | 228.47±4.39^Gc^ |
|  |  | 1:1 | 25.99±0.03^Da^ | 2.38±0.02^Da^ | 1.07±0.03^Ca^ | 7.73±0.22^Ed^ | 40.16±1.56^CDe^ | 7.15±0.06^De^ | 181.75±1.77^Ed^ |
|  |  | 1:5 | 25.98±0.02^Ca^ | 2.05±0.01^Eb^ | 0.98±0.02^Cb^ | 8.82±0.15^Ec^ | 48.83±0.53^Bc^ | 8.48±0.19^Cc^ | 222.68±3.34^Fc^ |
|  |  | 1:10 | 25.86±0.01^Dc^ | 1.70±0.02^Dc^ | 0.84±0.01^Cc^ | 9.84±0.28^Ea^ | 58.99±0.35^Cb^ | 10.07±0.24^Bb^ | 271.33±6.59^Fb^ |
|  |  | 1:20 | 25.81±0.03^Ed^ | 1.59±0.02^Dd^ | 0.77±0.02^Cd^ | 9.41±0.13^Eb^ | 69.85±0.40^Ca^ | 11.01±0.23^Ba^ | 360.91±6.02^Da^ |
|  | ACNs: EGCG | 1:0 | 25.93±0.03^Da^ | 1.43±0.02^Eb^ | 0.64±0.02^Ce^ | 9.54±0.18^Ec^ | 46.63±0.21^De^ | 7.95±0.14^Cd^ | 228.47±4.39^Ge^ |
|  |  | 1:1 | 25.68±0.01^Ed^ | 1.37±0.01^Dc^ | 0.78±0.02^Bb^ | 10.78±0.72^Eab^ | 54.37±0.53^Cd^ | 8.48±0.24^Bc^ | 243.53±4.82^Fd^ |
|  |  | 1:5 | 25.77±0.02^Cc^ | 1.25±0.02^Dd^ | 0.70±0.02^Bd^ | 11.41±0.28^Ea^ | 56.34±0.20^Dc^ | 9.57±0.26^Bb^ | 303.76±12.76^Fc^ |
|  |  | 1:10 | 25.69±0.03^Dd^ | 1.21±0.01^Ee^ | 0.73±0.00^Cc^ | 10.48±0.13^Db^ | 66.15±0.40^Cb^ | 9.85±0.45^Bb^ | 325.00±1.16^Gb^ |
|  |  | 1:20 | 25.84±0.02^Db^ | 1.70±0.02^Da^ | 0.91±0.02^Ba^ | 8.49±0.18^Ed^ | 71.81±0.00^Ca^ | 10.69±0.15^Aa^ | 401.45±4.18^Fa^ |
| 20d | ACNs: GA | 1:0 | 23.21±0.01^Ba^ | 1.63±0.02^Da^ | 0.00±0.02^Cb^ | 8.24±0.49^Fb^ | 44.90±0.18^Ae^ | 7.95±0.43^Bc^ | 275.14±5.93^Dd^ |
|  |  | 1:1 | 23.00±0.01^Cc^ | 1.56±0.01^Ec^ | 0.01±0.02^Cab^ | 8.34±0.34^Fab^ | 48.01±0.16^BCd^ | 8.01±0.15^Cc^ | 256.31±1.89^Ed^ |
|  |  | 1:5 | 23.13±0.04^Bb^ | 1.60±0.03^Db^ | -0.11±0.02^Dc^ | 8.68±0.19^Fab^ | 53.99±0.26^Cc^ | 9.28±0.25^Bb^ | 385.48±15.26^Ec^ |
|  |  | 1:10 | 23.01±0.01^Cc^ | 1.64±0.02^Da^ | -0.11±0.03^Dc^ | 8.52±0.19^Fab^ | 68.81±0.18^BCb^ | 10.18±0.41^Ba^ | 463.67±11.47^Eb^ |
|  |  | 1:20 | 22.94±0.01^Ed^ | 1.58±0.01^EFbc^ | 0.04±0.02^Ca^ | 8.91±0.28^Fa^ | 93.12±0.54^Aa^ | 10.57±0.76^Ca^ | 717.06±20.34^Ca^ |
|  | ACNs: QTI | 1:0 | 23.01±0.01^BCe^ | 1.55±0.02^Ee^ | 0.00±0.02^Cc^ | 9.12±0.23^Fd^ | 43.56±1.01^Be^ | 6.12±0.34^Cd^ | 245.23±10.89^Fd^ |
|  |  | 1:1 | 23.11±0.01^ABd^ | 1.64±0.01^Fd^ | 0.02±0.02^Bc^ | 9.46±0.41^Fcd^ | 47.93±0.78^Bd^ | 6.67±0.55^DEc^ | 263.27±20.29^Fd^ |
|  |  | 1:5 | 24.01±0.00^Ac^ | 2.68±0.03^Ac^ | -0.22±0.02^Dd^ | 9.92±0.29^Fbc^ | 55.26±0.45^Ac^ | 7.96±0.19^BCb^ | 303.81±11.66^Dc^ |
|  |  | 1:10 | 24.74±0.02^Bb^ | 3.50±0.01^DEb^ | 0.10±0.01^Bb^ | 10.29±0.27^Eab^ | 60.12±0.54^Ab^ | 8.51±0.29^Ba^ | 334.80±28.38^Db^ |
|  |  | 1:20 | 26.78±0.03^Ba^ | 5.50±0.02^Da^ | 1.06±0.02^Ba^ | 10.74±0.07^Ea^ | 71.84±1.50^Aa^ | 8.83±0.20^Ba^ | 380.84±8.69^Ca^ |
|  | ACNs: RUT | 1:0 | 25.68±0.02^Fb^ | 1.42±0.02^Ea^ | 0.75±0.02^Bc^ | 8.35±0.49^Fc^ | 47.86±0.19^Ce^ | 8.61±0.20^Bd^ | 252.99±6.13^Ec^ |
|  |  | 1:1 | 25.60±0.03^Ed^ | 1.37±0.02^Eb^ | 0.87±0.01^Ba^ | 10.09±0.28^Fa^ | 49.20±0.19^Bd^ | 9.82±0.05^Ba^ | 320.95±9.36^Da^ |
|  |  | 1:5 | 25.69±0.01^Eb^ | 1.35±0.02^Fb^ | 0.75±0.01^Bc^ | 9.73±0.52^Fab^ | 56.40±0.19^Bc^ | 9.74±0.07^Bab^ | 275.38±12.39^Db^ |
|  |  | 1:10 | 25.65±0.01^Fc^ | 1.25±0.01^Ec^ | 0.77±0.02^Bbc^ | 9.35±0.17^Fb^ | 52.13±0.34^Db^ | 8.90±0.19^Bc^ | 306.66±4.82^Da^ |
|  |  | 1:20 | 25.72±0.01^Fa^ | 1.25±0.03^Fc^ | 0.77±0.01^Bb^ | 8.89±0.43^Fbc^ | 60.23±0.34^ABa^ | 9.53±0.12^Bb^ | 310.52±7.88^Da^ |
|  | ACNs: CAT | 1:0 | 25.68±0.02^Fd^ | 1.42±0.02^Ee^ | 0.75±0.02^Be^ | 8.35±0.49^Fa^ | 47.86±0.19^Cd^ | 8.61±0.20^Bd^ | 252.99±6.13^Ec^ |
|  |  | 1:1 | 25.82±0.01^Ec^ | 2.40±0.01^Da^ | 1.21±0.02^Aa^ | 7.01±0.49^EFc^ | 42.12±0.19^Ce^ | 7.72±0.12^Be^ | 179.63±4.68^Ee^ |
|  |  | 1:5 | 25.88±0.02^Eb^ | 2.07±0.00^Db^ | 0.99±0.02^BCb^ | 7.85±0.43^Fbc^ | 53.25±0.19^Ac^ | 9.01±0.09^Bc^ | 242.56±1.16^Ed^ |
|  |  | 1:10 | 25.84±0.00^Ec^ | 1.62±0.02^Ec^ | 0.82±0.01^Dc^ | 8.78±0.39^Fa^ | 62.14±0.19^Bb^ | 10.60±0.18^Ab^ | 303.96±1.16^Eb^ |
|  |  | 1:20 | 25.91±0.01^Da^ | 1.49±0.02^Ed^ | 0.79±0.02^Bd^ | 8.10±0.16^Fab^ | 73.83±0.70^Ba^ | 10.93±0.03^Ba^ | 406.28±5.71^Ca^ |
|  | ACNs: EGCG | 1:0 | 25.68±0.02^Fb^ | 1.42±0.02^Eb^ | 0.75±0.02^Bc^ | 8.35±0.49^Fc^ | 47.86±0.19^Ce^ | 8.61±0.20^Bc^ | 252.99±6.13^Ee^ |
|  |  | 1:1 | 25.60±0.02^Fd^ | 1.28±0.02^Ec^ | 0.80±0.02^Bb^ | 9.69±0.46^Fb^ | 54.04±0.19^Cd^ | 9.78±0.28^Ab^ | 292.76±3.72^Dd^ |
|  |  | 1:5 | 25.68±0.02^Db^ | 1.16±0.02^Ed^ | 0.71±0.02^ABd^ | 10.30±0.27^Fa^ | 61.12±0.19^Cc^ | 9.71±0.15^Bb^ | 344.11±4.82^Dc^ |
|  |  | 1:10 | 25.63±0.02^Ec^ | 1.26±0.02^Cc^ | 0.80±0.01^Bb^ | 9.85±0.26^Db^ | 73.49±0.19^Ab^ | 8.84±0.25^DEc^ | 383.50±5.71^Eb^ |
|  |  | 1:20 | 25.95±0.01^Ca^ | 1.46±0.02^Ea^ | 0.96±0.01^Aa^ | 7.43±0.45^Fd^ | 83.05±0.78^Aa^ | 10.63±0.10^Aa^ | 414.00±8.11^Ea^ |
| 24d | ACNs: GA | 1:0 | 23.30±0.03^Aa^ | 1.59±0.03^Ea^ | 0.00±0.01^Cc^ | 6.43±0.17^Gc^ | 36.60±0.33^Ce^ | 8.31±0.05^ABc^ | 328.28±1.63^Bd^ |
|  |  | 1:1 | 23.06±0.01^Bc^ | 1.50±0.01^Fb^ | 0.02±0.01^Cbc^ | 6.57±0.11^Gbc^ | 38.31±0.13^Ed^ | 7.80±0.08^Cd^ | 318.14±8.68^Dd^ |
|  |  | 1:5 | 23.12±0.01^Bb^ | 1.59±0.03^Da^ | -0.03±0.03^Bd^ | 6.72±0.26^Gbc^ | 48.01±0.51^Ec^ | 8.40±0.07^Dc^ | 451.07±6.27^Bc^ |
|  |  | 1:10 | 23.08±0.01^Ac^ | 1.60±0.01^Ea^ | 0.04±0.0^Bb^ | 7.31±0.09^Gab^ | 63.24±0.25^Eb^ | 10.47±0.53^ABb^ | 540.56±20.07^BCb^ |
|  |  | 1:20 | 22.96±0.01^Dd^ | 1.57±0.01^Fa^ | 0.07±0.02^Ba^ | 7.56±0.25^Ga^ | 77.35±0.69^Da^ | 12.16±0.16^Aa^ | 790.76±9.89^Aa^ |
|  | ACNs: QTI | 1:0 | 23.02±0.03^Be^ | 1.53±0.03^Ee^ | 0.01±0.01^Cc^ | 7.87±0.39^Gc^ | 35.78±0.65^De^ | 5.86±0.10^Dd^ | 326.56±5.89^Ce^ |
|  |  | 1:1 | 23.07±0.01^Cd^ | 1.60±0.01^Gd^ | 0.03±0.02^ABc^ | 8.18±0.18^Gc^ | 38.64±0.78^Cd^ | 6.31±0.14^Dcd^ | 341.32±1.46^Cd^ |
|  |  | 1:5 | 23.86±0.04^Bc^ | 2.45±0.06^Cc^ | -0.14±0.02^Cd^ | 8.39±0.37^Gbc^ | 42.80±0.13^Ec^ | 6.65±0.78^Dc^ | 360.43±8.89^Cc^ |
|  |  | 1:10 | 24.79±0.01^Ab^ | 3.45±0.02^Eb^ | 0.15±0.02^Ab^ | 8.79±0.17^Fab^ | 48.73±0.62^Db^ | 7.74±0.39^Db^ | 398.08±4.57^Bb^ |
|  |  | 1:20 | 27.12±0.04^Aa^ | 5.54±0.03^Da^ | 1.12±0.03^Aa^ | 9.21±0.55^Fa^ | 61.00±0.25^Da^ | 8.74±0.22^BCa^ | 416.61±4.05^Ba^ |
|  | ACNs: RUT | 1:0 | 25.62±0.01^Gd^ | 1.35±0.02^Fa^ | 0.86±0.01^Ab^ | 7.41±0.32^Gc^ | 50.09±0.23^Bd^ | 9.24±0.38^Ab^ | 241.79±0.67^Fc^ |
|  |  | 1:1 | 25.56±0.01^Fe^ | 1.29±0.02^Fb^ | 0.92±0.01^Aa^ | 9.15±0.20^Ga^ | 49.15±1.29^Bd^ | 10.11±0.21^Aa^ | 278.47±2.68^Fb^ |
|  |  | 1:5 | 25.75±0.02^Da^ | 1.22±0.02^Gd^ | 0.83±0.02^Ac^ | 8.84±0.27^Gab^ | 54.64±1.21^Cc^ | 10.23±0.06^Aa^ | 277.32±4.07^Db^ |
|  |  | 1:10 | 25.64±0.01^Gc^ | 1.24±0.01^Ec^ | 0.86±0.02^Ab^ | 8.49±0.78^Fab^ | 57.99±0.23^Bb^ | 10.17±0.22^Aa^ | 285.42±9.29^Eb^ |
|  |  | 1:20 | 25.71±0.01^Fb^ | 1.17±0.01^Ge^ | 0.85±0.01^Abc^ | 8.04±0.59^Fbc^ | 62.55±0.46^Aa^ | 10.11±0.06^Aa^ | 307.43±0.67^Da^ |
|  | ACNs: CAT | 1:0 | 25.62±0.01^Gb^ | 1.35±0.02^Fe^ | 0.86±0.01^Ae^ | 7.41±0.32^Gab^ | 50.09±0.23^Bd^ | 9.24±0.38^Ad^ | 241.79±0.67^Fd^ |
|  |  | 1:1 | 25.75±0.00^Fa^ | 2.23±0.02^Fa^ | 1.19±0.01^Aa^ | 6.43±0.08^Fc^ | 50.36±0.61^Ad^ | 8.17±0.13^Ae^ | 201.63±2.01^De^ |
|  |  | 1:5 | 25.62±0.01^Fb^ | 2.05±0.01^Eb^ | 1.12±0.02^Ab^ | 7.00±0.27^Gbc^ | 54.24±0.80^Ac^ | 9.96±0.07^Ac^ | 246.43±2.68^Ec^ |
|  |  | 1:10 | 25.60±0.01^Gc^ | 1.60±0.02^Ec^ | 1.00±0.02^Ac^ | 7.90±0.26^Ga^ | 63.89±0.40^Ab^ | 10.64±0.26^Ab^ | 309.36±3.06^Eb^ |
|  |  | 1:20 | 25.75±0.00^Fa^ | 1.43±0.01^Fd^ | 0.89±0.01^Ad^ | 7.30±0.64^Gab^ | 77.29±0.84^Aa^ | 13.21±0.14^Aa^ | 380.02±1.16^Da^ |
|  | ACNs: EGCG | 1:0 | 25.62±0.01^Gd^ | 1.35±0.02^Fb^ | 0.86±0.01^Ab^ | 7.41±0.32^Gc^ | 50.09±0.23^Be^ | 9.24±0.38^Ac^ | 241.79±0.67^Fe^ |
|  |  | 1:1 | 25.68±0.00^Eb^ | 1.26±0.02^Ec^ | 0.88±0.01^Ab^ | 8.81±0.44^Fb^ | 57.32±0.46^Bd^ | 9.74±0.16^Ab^ | 267.28±8.54^Ed^ |
|  |  | 1:5 | 25.63±0.01^Ec^ | 1.15±0.02^Ed^ | 0.73±0.02^Ad^ | 9.59±0.33^Ga^ | 66.44±0.93^Ac^ | 10.68±0.07^Aa^ | 324.42±2.01^Ec^ |
|  |  | 1:10 | 25.49±0.01^Fe^ | 1.11±0.01^Fe^ | 0.83±0.01^Ac^ | 9.32±0.09^Dab^ | 74.21±1.01^Ab^ | 10.37±0.09^Aa^ | 348.36±5.94^Fb^ |
|  |  | 1:20 | 25.81±0.01^Da^ | 1.45±0.03^Ea^ | 0.98±0.01^Aa^ | 6.58±0.21^Gd^ | 79.97±1.06^Ba^ | 10.72±0.22^Aa^ | 389.68±5.71^Ga^ |

**Fig. S1**. Mass spectrograms of HPLC-ESI-QTOF-MS^2^ for qualitative phenolic compounds of BHJ.


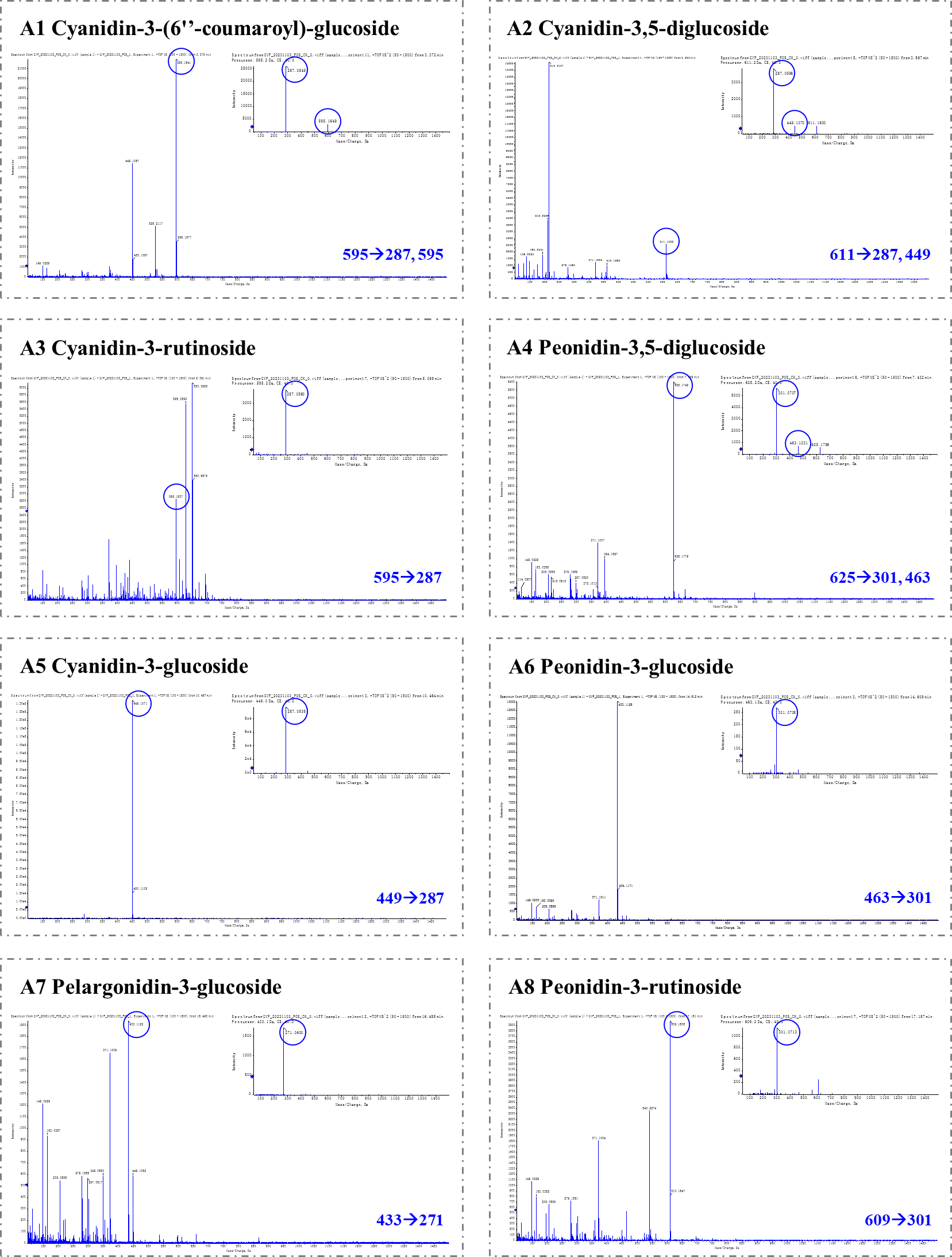


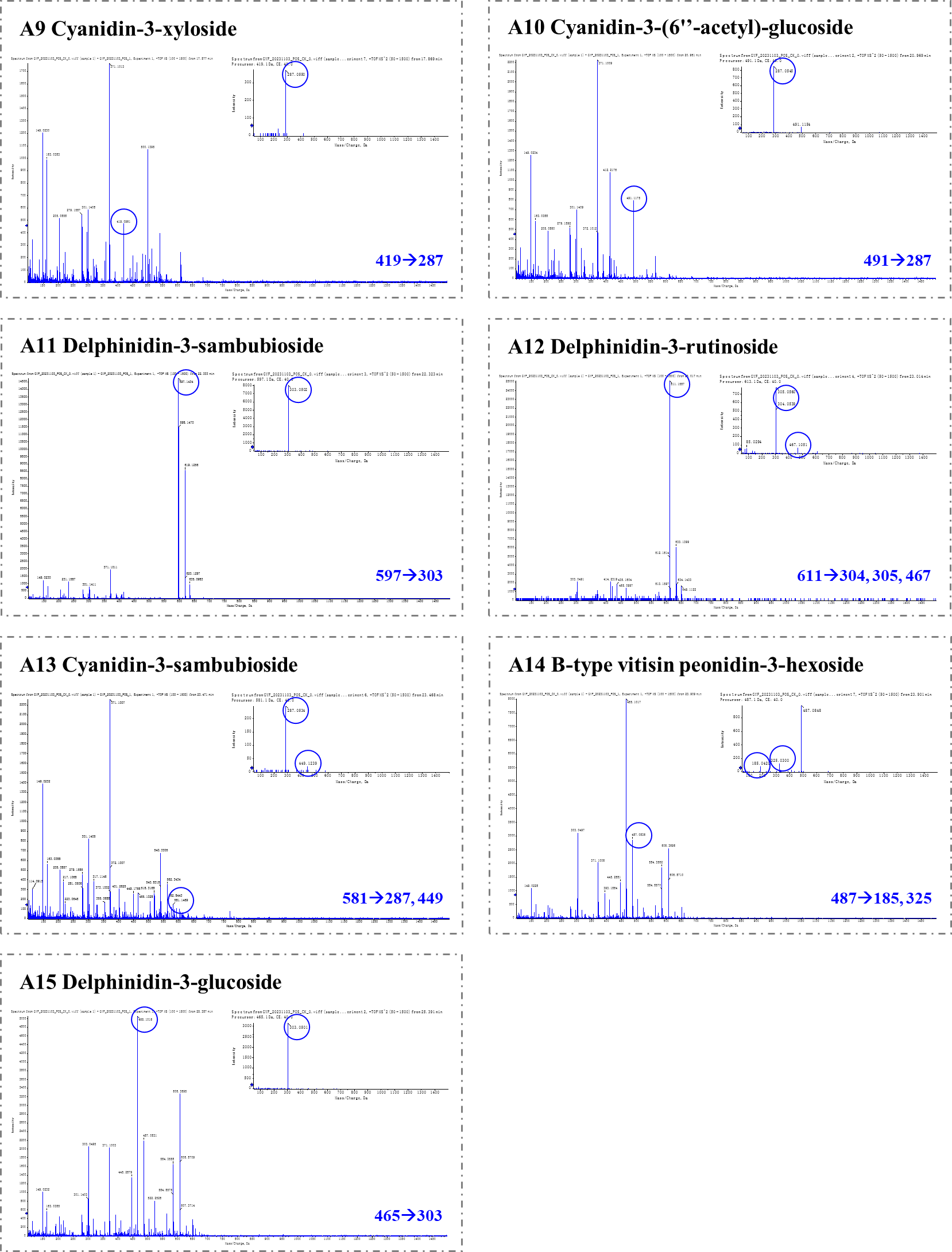


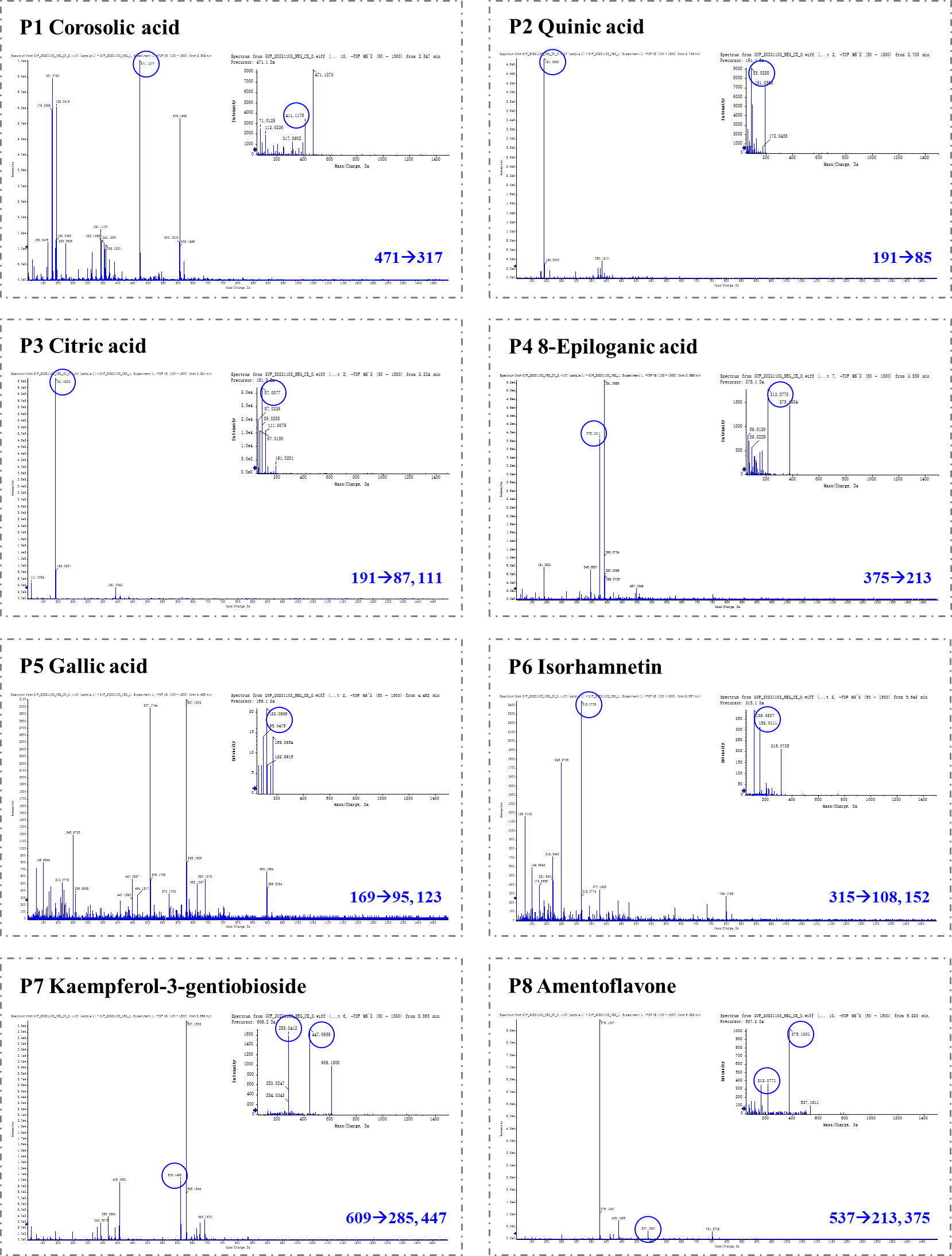


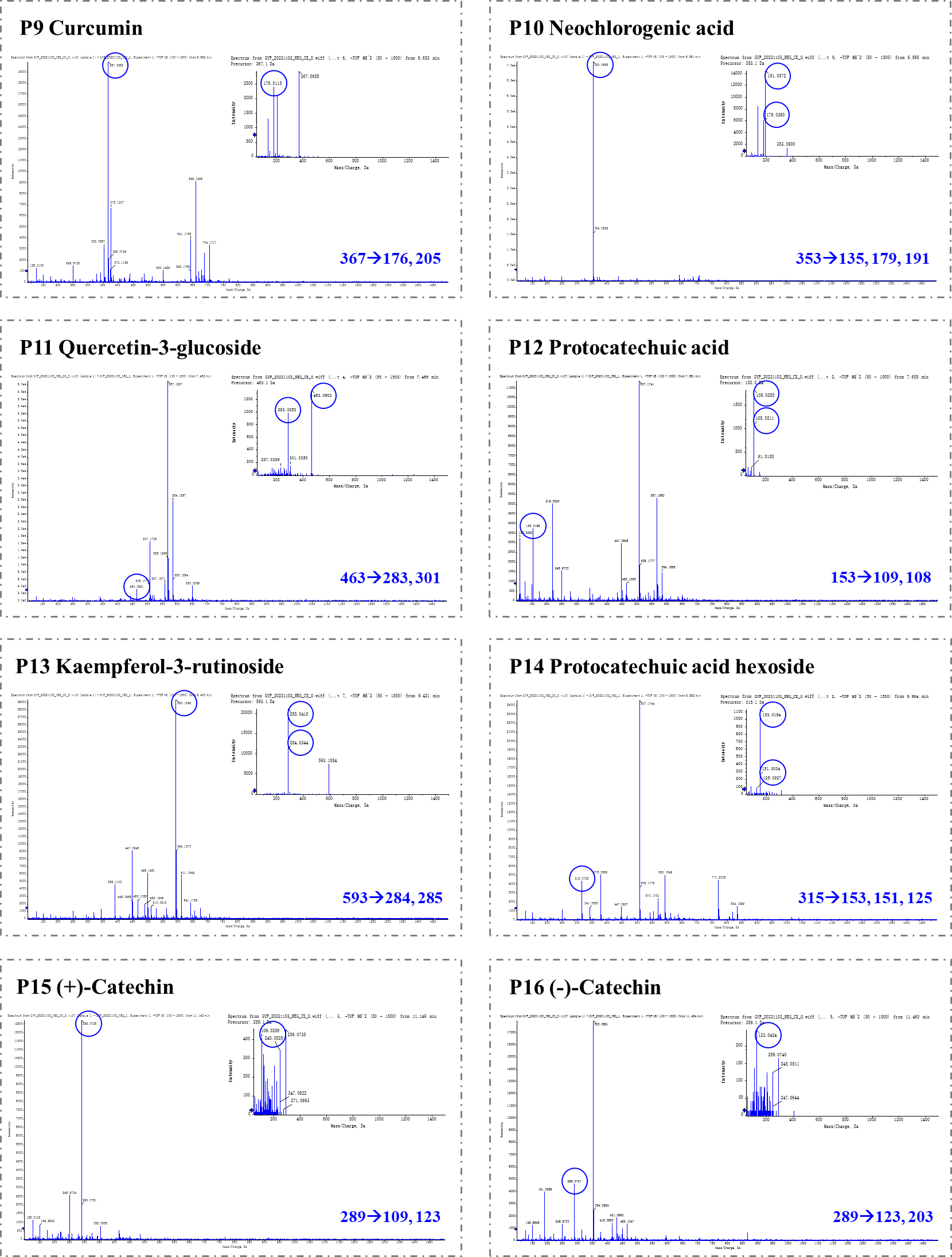


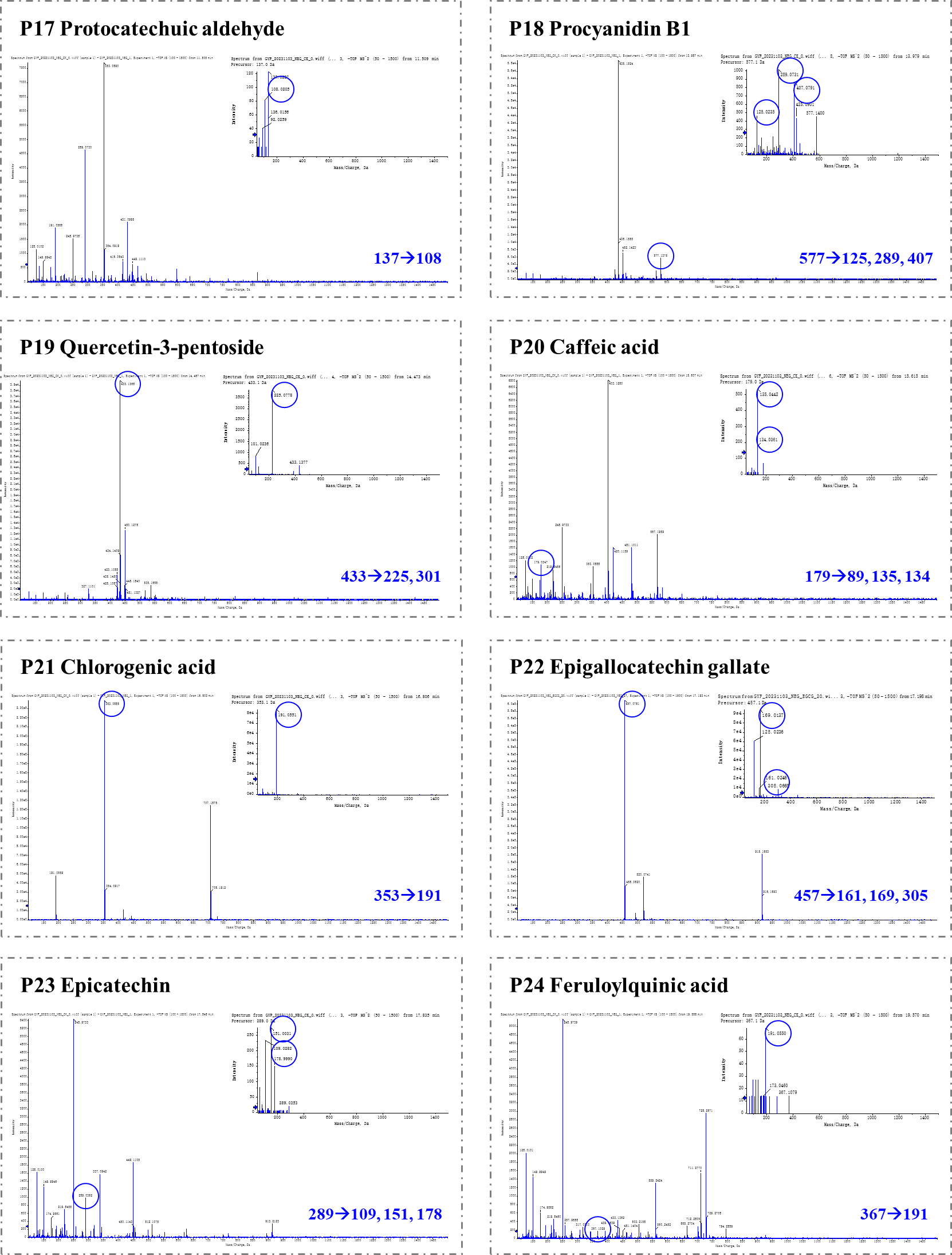


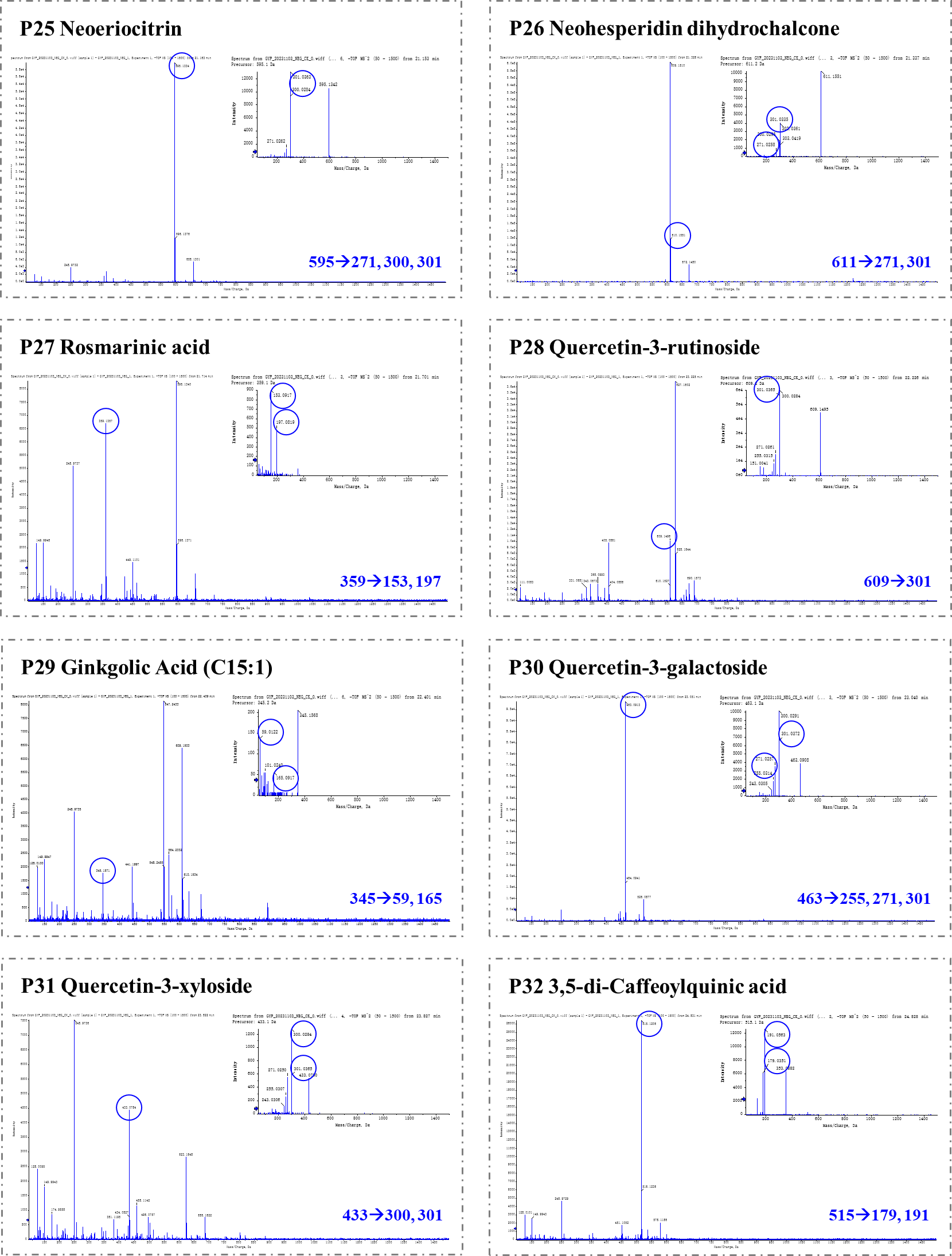


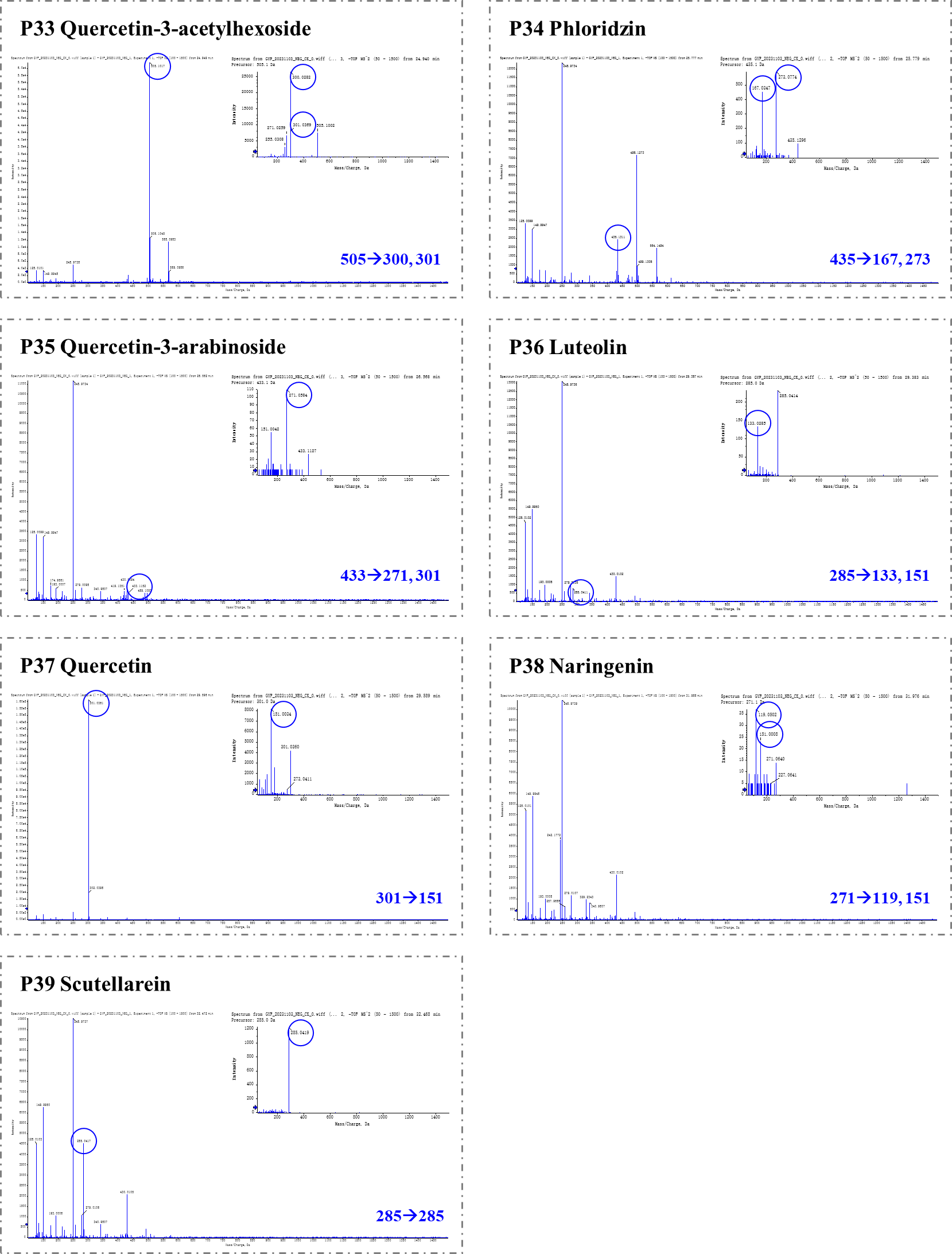

Supplement: Supplementary file 1 — Table S1. HPLC-ESI-QTOF-MS2 of phenolic compounds of BHJ. [file mmc1.docx]
